# A data driven change-point epidemic model for assessing the impact of large gathering and subsequent movement control order on COVID-19 spread in Malaysia

**DOI:** 10.1371/journal.pone.0252136

**Published:** 2021-05-27

**Authors:** Sarat C. Dass, Wai M. Kwok, Gavin J. Gibson, Balvinder S. Gill, Bala M. Sundram, Sarbhan Singh

**Affiliations:** 1 School of Mathematical and Computer Sciences, Heriot-Watt University Malaysia, Putrajaya, Wilayah Persekutuan Putrajaya, Malaysia; 2 School of Mathematical and Computer Sciences, Heriot-Watt University, Edinburgh, Scotland, United Kingdom; 3 Institute for Medical Research, Ministry of Health, Shah Alam, Selangor, Malaysia; Texas A&M University College Station, UNITED STATES

## Abstract

The second wave of COVID-19 in Malaysia is largely attributed to a four-day mass gathering held in Sri Petaling from February 27, 2020, which contributed to an exponential rise of COVID-19 cases in the country. Starting from March 18, 2020, the Malaysian government introduced four consecutive phases of a Movement Control Order (MCO) to stem the spread of COVID-19. The MCO was implemented through various non-pharmaceutical interventions (NPIs). The reported number of cases reached its peak by the first week of April and then started to reduce, hence proving the effectiveness of the MCO. To gain a quantitative understanding of the effect of MCO on the dynamics of COVID-19, this paper develops a class of mathematical models to capture the disease spread before and after MCO implementation in Malaysia. A heterogeneous variant of the Susceptible-Exposed-Infected-Recovered (SEIR) model is developed with additional compartments for asymptomatic transmission. Further, a change-point is incorporated to model disease dynamics before and after intervention which is inferred based on data. Related statistical analyses for inference are developed in a Bayesian framework and are able to provide quantitative assessments of (1) the impact of the Sri Petaling gathering, and (2) the extent of decreasing transmission during the MCO period. The analysis here also quantitatively demonstrates how quickly transmission rates fall under effective NPI implementation within a short time period. The models and methodology used provided important insights into the nature of local transmissions to decision makers in the Ministry of Health, Malaysia.

## Introduction

Imported cases from China contributed to the first COVID-19 wave in Malaysia from January 25, 2020 to February 26, 2020 [1]. This first wave had a total of 22 cases out of which 20 were directly linked to foreign travel while the remaining two cases were local transmissions [2, 3]. The second wave of COVID-19 in Malaysia was largely attributed to a four-day mass gathering held in Sri Petaling from February 27, 2020, which contributed to an exponential rise of COVID-19 cases in the country. This gathering involved over 16, 000 participants including a large number of foreign visitors from countries that later registered COVID-19 cases; see, for example, [4] which refers to the official Facebook link of the Ministry of Health, Malaysia [5]. On March 18, 2020, the Malaysian government introduced a nationwide lockdown which was the Phase 1 Movement Control Order (MCO) throughout the country to stem the spread of COVID-19. Phase 1 MCO was enforced for a 2-week period starting from March 18, 2020 to March 31, 2020. During this phase, various non-pharmaceutical interventions (NPIs) were strictly enforced by means of movement restrictions, wearing of face masks, social distancing and hand hygiene practices to reduce disease transmission. The Phase I MCO was then evaluated after 2 weeks based on case trends and model forecasts by the Ministry of Health (MOH) Malaysia. As local transmission persisted, the MCO was extended a total of four times until May 12, 2020. Phases 2, 3 and 4 of the MCO covered periods starting from April 1, 2020 until May 12, 2020 (Phase 2—April 1, 2020 to April 14, 2020, Phase 3—April 15th to April 30, 2020, and Phase 4—May 1, 2020 to May 12, 2020). Subsequently, from May 4, 2020, the MCO was eased into the Conditional MCO (CMCO) until June 9, 2020. However, during CMCO, there were still several identified hot-spots of COVID-19 which were placed under Enhanced MCO (EMCO) with the aforementioned strict movement control restrictions.

The implementation of MCO proved to be effective—the reported COVID-19 cases reached its peak around the first week of April and subsequently started to reduce. However, concerns remained whether a rebound in transmission would occur when the MCO was lifted and if compliance to NPIs were not followed strictly at that time. In order to gain a quantitative understanding of the effect of MCO, we developed a class of mathematical models to capture the dynamics of COVID-19 spread before and after the MCO implementation. A variant of the Susceptible-Exposed-Infected-Recovered (SEIR) model is proposed and developed in this paper which incorporates heterogeneity in the transmission dynamics, additional compartments for asymptomatic transmission and a change-point, chosen adaptively based on data, to reflect the shift in spread dynamics after the MCO implementation. The models developed in this paper are able provide a quantitative assessment of the extent of COVID-19 spread during the pre-MCO (large gathering) and MCO periods by means of a measure of infectivity developed from them. This measure is similar to the basic reproduction number, commonly denoted by *ℛ*_0_, but can be calculated for more complex epidemic models such as the ones proposed here.

Deterministic compartmental models, such as the Susceptible-Infected-Recovered (SIR) or the Susceptible-Exposed-Infectious-Recovered (SEIR) models, provide a good theoretical framework to study infectious disease spread, and have been widely used and reported in the literature. However, more complex versions of these models, and their stochastic counterparts require data-rich inputs to model all aspects of the disease dynamics. Data-rich inputs, if lacking, can be compensated using reliable and informative prior elicitation. Considering the acute nature and scale of the pandemic as well as the urgent need for a multisectorial response, comprehensive data availability of the pandemic was limited in Malaysia. For example, the open source website outbreak.my initially reported a transmission network for all cases; however, it was unable to cope with the scale of the pandemic when it intensified. Factoring in this data limitation, we choose to develop models that are deterministic, rather than stochastic, while ensuring that they are able to capture salient transmission dynamics satisfactorily. As mentioned earlier, we enhance the deterministic models by incorporating compartments for asymptomatic transmission and a change-point to reflect the shift in disease dynamics. We also take into account heterogeneity in the disease spread such as varying contacts among susceptibles within the closed population. The starting point of our proposed models are the class of epidemic models with power transmission dynamics which are shown to incorporate heterogeneity (see [6, 7]).

Several studies in the literature [8–12] have analyzed the effects of NPIs in reducing the number of COVID-19 cases. In [8], the effects of different types of NPIs on COVID-19 cases are modeled using a negative-binomial distribution whose underlying parameters incorporate country information, type of NPI implemented and change-point effects. The associated Bayesian analysis is carried out using Markov Chain Monte Carlo (MCMC) algorithms to arrive at posterior parameter estimates and credible intervals. No epidemic models are considered in this work. A generalization of the SEIR epidemic model is considered in [10] to understand the dynamics of transmission in New York, USA, under various NPI settings. However, the model is complex and requires data-rich inputs for the estimation of all unknown parameters. As a result, the authors derive baseline epidemiological parameters from published literature and not from actual observed cases in New York, and in the end conduct a simulation study based on the assumed parameter values. The study in [9] extends the work of [13] and computes a time-varying basic reproduction number as a way of gauging the effect of NPIs over time. Both these works assume that serial intervals (i.e., the time between onset of symptoms for the infector and the infectee) can be computed for each case, which is another situation requiring data-rich input.

Studies that use compartmental epidemic models as a way of gauging the time-varying effects of NPIs have emerged over the course of the pandemic [14–16]. Compartmental epidemic models naturally model disease spread via contact rates which directly quantify the extent of NPIs (since, as mentioned earlier, NPIs are designed to reduce person-to-person transmission). Thus, epidemic models provide a natural approach for considering time-varying effects of the MCO period. Further, in this paper, the estimation of SEIR parameters is carried out based on local considerations and local data; they are not obtained from published literature based on studies conducted elsewhere where their local dynamics can be vastly different.

We seek to address one important aspect of Malaysia’s multifaceted response to the COVID-19 pandemic, that is, to inform the health officials at MOH and aid them in their decision-making. Thus, our model was developed under local considerations using local data. Our model and related analyses are able to provide a quantitative assessment of (1) the impact of the Sri Petaling gathering, and (2) the extent of decreasing transmission during the MCO period by incorporating a time-varying contact rate parameter, which is estimated using locally available data. In essence, the proposed models here are being used as a lens to interpret the observed data in terms of when, and to what extent, a reduction in COVID-19 transmission occurred as result of the implementation of MCO.

## Materials and methods

### Data collection

**Fig 1:**
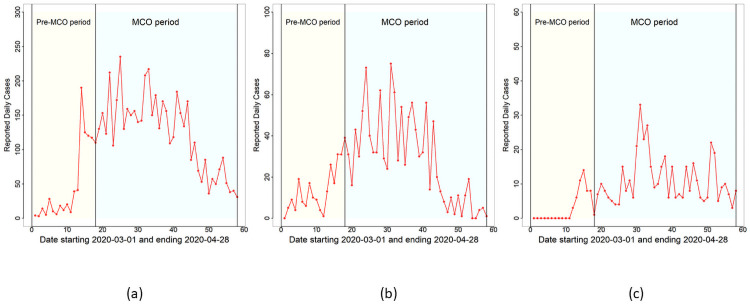
Reported daily cases. Reported daily cases for (a) Malaysia, (b) Selangor and (c) Sarawak. The time period considered is from March 1, 2020 to April 28, 2020.

Daily situation reports on COVID-19 cases in Malaysia are published by the National Crisis Preparedness and Response Centre (CPRC) of MOH, as well as other official websites (such as outbreak.my). The data on daily COVID-19 cases have been published since 21 January 2020 and are publicly available. The reports consist of confirmed daily and cumulative cases, recovered cases and deaths, as well as cases requiring ICU care and ventilator support. Cases by states are also available for the 13 states and 3 federal territories. In this study, we studied characteristics of the second COVID-19 wave in Malaysia starting from March 1, 2020 (corresponding to the final day of the Sri Petaling gathering). Data used for the current study are confirmed daily cases for Malaysia, and for two states: Selangor and Sarawak. These states were chosen to illustrate the aggressive transmission propagated by the Sri Petaling gathering. Selangor is the state where Sri Petaling is located and from where a majority of the participants originated, whereas Sarawak represents a state which was essentially not affected by this gathering. The time period of study is between March 1, 2020 (end of Sri Petaling gathering) and April 28, 2020, covering the period immediately after the Sri Petaling gathering and the first three phases of the MCO. Our study duration is further divided into two periods. The first period ranges from March 1, 2020 until March 18, 2020, which covers the subsequent 17 days after the gathering. The second period is taken from March 18, 2020 until April 28, 2020, covering the three successive Phases 1, 2 and 3 of the MCO. Fig 1 gives the trajectories of reported daily COVID-19 cases between March 1, 2020 and April 28, 2020 for Malaysia, and the states of Selangor and Sarawak.

All COVID-19 cases reported by MOH were confirmed by real-time reverse transcriptase-polymerase chain reaction (real-time RT-PCR) tests. A positive case was reported when the person in question was found to be positive for SARS-CoV-2 via a real-time RT-PCR test. Upon confirmation, the individual was isolated at COVID-19 designated hospitals and healthcare facilities [17]. Active cases are defined as infected persons who were currently undergoing treatment, and hence, isolated and removed; the individual is assumed to be unable to infect other susceptibles in the population, and hence “removed” from further modelling steps. This study did not consider transmission from positive isolated COVID-19 patients to health personnel as there was no evidence of this type of transmission occurring in the COVID-19 designated hospitals in Malaysia.

### The SEIR model

The typical and well-known SEIR compartmental model consists of four compartments (Susceptible, Exposed, Infected and Recovered) representing different stages of evolution of an infectious disease, such as COVID-19, in a population. Susceptible individuals come in contact with one or more infected individuals in the population, and subsequently, become exposed to the virus. The virus then incubates within these individuals for some time. At the end of the incubation period, the exposed person becomes infectious and transmits the disease to other susceptibles in the population who come in close contact to him/her. The infected person is assumed to be infectious for a certain period (called the infectious period) after which the person recovers, dies or becomes immune. The deterministic SEIR model is given by a set of nonlinear ordinary differential equations (ODEs):

$$\begin{array}{ccc} \dot{S}\left(t\right) & = & -h(S(t),I(t)) \end{array}$$ (1)

$$\begin{array}{ccc} \dot{E}\left(t\right) & = & h(S(t),I(t))-\delta \;E(t) \end{array}$$ (2)

$$\begin{array}{ccc} \dot{I}\left(t\right) & = & \delta \;E(t)-\gamma \;I(t),\;\;\;\text{and} \end{array}$$ (3)

$$\begin{array}{ccc} \dot{R}\left(t\right) & = & \gamma \;I(t) \end{array}$$ (4)

where *S*(*t*), *E*(*t*), *I*(*t*) and *R*(*t*) represents, respectively, the susceptible, exposed, infected and recovered compartments representing the total number of individuals in each compartment at time *t* (here, $\dot{x}\left(t\right)$ denotes the derivative of *x*(*t*) with respect to time *t* for *x* ∈ {*S*, *E*, *I*, *R*}), *N* is the total population size, and $h\left(S,I\right)=\beta \;\frac{S}{N}\;I$ is the rate of new infections (or, the number of new cases in the population). The parameters that govern the trajectory of the SEIR model are *θ* ≡ (*β*, *δ*, *γ*, *i*_0_, *e*_0_) which are, respectively, the transmission rate (i.e., number of individuals in the population an infected person comes in contact with and successfully transmits the disease per unit time), the rate of incubation of the disease, the rate of infectiousness, the initial number of infectives and the initial number of exposed individuals. Since $\dot{S}\left(t\right)+\dot{E}\left(t\right)+\dot{I}\left(t\right)+\dot{R}\left(t\right)=0$, it follows that *S*(*t*) + *E*(*t*) + *I*(*t*) + *R*(*t*) = *N* for all *t*. Reparametrizing *S*(*t*) = *S*(*t*)/*N*, *E*(*t*) = *E*(*t*)/*N*, *I*(*t*) = *I*(*t*)/*N* and *R*(*t*) = *R*(*t*)/*N*, the renormalized versions of *S*, *E*, *I* and *R* represent the proportion, rather than the total number of individuals, in each compartment. In the renormalized SEIR model, *S*(*t*) + *E*(*t*) + *I*(*t*) + *R*(*t*) = 1 and the rate of new infections become

$$\begin{array}{c} h(S,I)=\beta \;S\;I. \end{array}$$ (5)

Based on initial values of $S\left(0\right)\equiv s_{0}=1-\frac{i_{0}}{N}-\frac{e_{0}}{N}$, $E\left(0\right)=\frac{e_{0}}{N}$, $I\left(0\right)=\frac{i_{0}}{N}$, and *R*(0) = 0 at time *T*_0_ = 0, the SEIR ODE system can be solved numerically to yield the values of *S*(*t*), *E*(*t*), *I*(*t*) and *R*(*t*) for every *t* ∈ [*T*_0_, *T*_1_] where *T*_1_ denotes the final time-point. In (1)–(4), the incubation period, 1/*δ*, is the reciprocal of the incubation rate *δ*, and similarly, the infectious period 1/*γ* is the reciprocal of the infectious rate *γ*. The correspondence between rates and exponential sojourn times is only approximately so since it is not so straightforward to establish this correspondence with an individual-based stochastic model given the non-linear nature of the process.

Modifications to the SEIR formulation (1)–(4) are made to adapt it to COVID-19 in Malaysia. The last compartment of the SEIR model in this case should be “Removed”, and not “Recovered”, representing all infectious individuals who are effectively isolated following a positive test result. In Malaysia, such patients are isolated in hospital wards to avoid further contacts with susceptibles [17]. For COVID-19 in particular, the onset of symptoms does not necessarily indicate the start of infectiousness; in fact, the onset of infectiousness may be somewhat earlier. Thus, the infectious period 1/*γ* represents the period of effective infectiousness, that is, the period from the start of infectiousness (asymptomatic or symptomatic) until the individual is isolated and can no longer infect others. Based on this understanding, 1/*δ* represents the incubation period, which is the period starting from getting infected until the onset of infectiousness. Recent studies on COVID-19 have also reported growing evidence of asymptomatic infections [18–20] which the original SEIR model does not incorporate. Hence, a new modified SEIR model is developed in the next section to account for isolated patients and asymptomatic transmissions.

Observed data in Malaysia consists of the total number of confirmed daily cases as reported by outbreak.my and CPRC (i.e., the number of cases that were tested positive, and hence, effectively isolated). Hence, only the *R* compartment of the SEIR model can be related to observed data while other compartments of the SEIR model remain unobserved. In the subsequent model development, the *R* compartment is further split into two: observed and asymptomatic sub-compartments corresponding to reported and asymptomatic infections; details are provided in the next section. An additional assumption made is that individuals from the *R* compartment do not return to the *S* compartment—at least on the timescales over which the epidemic is observed; see, for example, [21, 22].

In order to provide a quantitative assessment of the impact of the MCO, we modify (1)–(4) to incorporate an instantaneous time-varying transmission rate, *β* ≡ *β*(*t*) (see [23], for example) which is able to quantify the extent of disease transmission at time *t*. The MCO can be deemed effective if the function *β*(*t*) shows a decay reflecting an increasing effectiveness in reducing transmission among individuals over time due to implementation of the NPIs.

### The modified SEIR model

The aforementioned SEIR model does not account for heterogeneity, asymptomatic transmission and change points. To this end, we propose models with power transmission dynamics that incorporate heterogeneity in the disease parameters; see, for example, [6, 7]. Further, the infectious compartment in (3) of the SEIR model is now split into two, *I*_*o*_ and *I*_*c*_, for symptomatic (or, **o**bserved) and asymptomatic (or, **c**ryptic) individuals, who, respectively, exhibit and do not (or, mildly) exhibit symptoms but are nevertheless infectious. Correspondingly, the *R* compartment is also split into two, as mentioned earlier, to accommodate quarantined and un-quarantined cases. It is assumed that the proportion of individuals transiting from *E* to *I*_*o*_ is *p*. The remaining exposed individuals (a proportion of 1 − *p*) transition into the *I*_*c*_ compartment and remain undetected throughout their disease experience.

**Fig 2:**
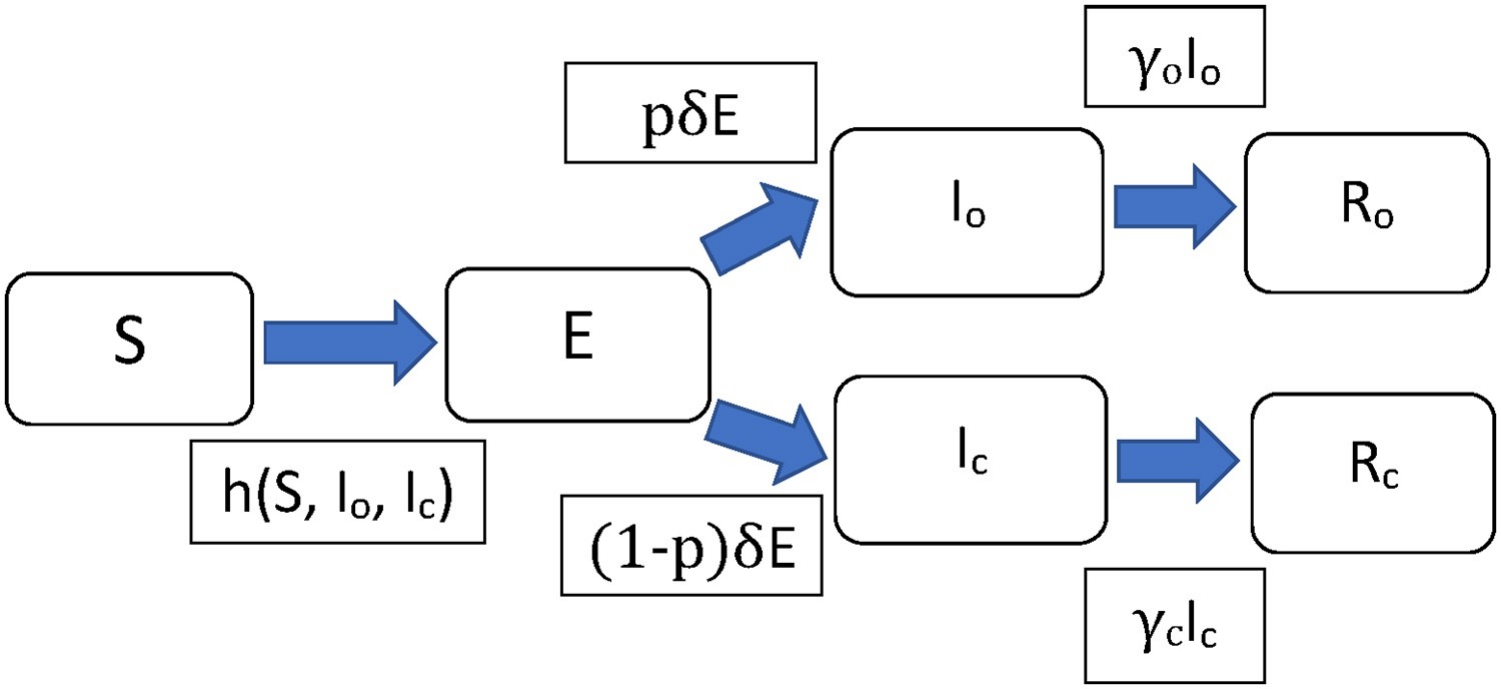
Flow chart of the modified SEIR model. Compartments of the modified SEIR model and their associated parameters.

From now on, we consider only renormalized state values which represent proportions, and not actual numbers, of the population. The modified SEIR model with asymptomatic and observed infectiousness is given by the following system of ODEs:

$$\begin{array}{ccc} \dot{S}\left(t\right) & = & -h_{}\left(S\left(t\right),I_{o}\left(t\right),I_{c}\left(t\right)\right) \end{array}$$ (6)

$$\begin{array}{ccc} \dot{E}\left(t\right) & = & h_{}\left(S\left(t\right),I_{o}\left(t\right),I_{c}\left(t\right)\right)-\delta \;E\left(t\right) \end{array}$$ (7)

$$\begin{array}{ccc} {\dot{I}}_{o}\left(t\right) & = & p\;\delta \;E\left(t\right)-\gamma_{o}\;I_{o}\left(t\right) \end{array}$$ (8)

$$\begin{array}{ccc} {\dot{R}}_{o}\left(t\right) & = & \gamma_{o}\;I_{o}\left(t\right) \end{array}$$ (9)

$$\begin{array}{ccc} {\dot{I}}_{c}\left(t\right) & = & \left(1-p\right)\;\delta \;E\left(t\right)-\gamma_{c}\;I_{c}\left(t\right) \end{array}$$ (10)

$$\begin{array}{ccc} {\dot{R}}_{c}\left(t\right) & = & \gamma_{c}\;I_{c}\left(t\right) \end{array}$$ (11)

where the rate of new infections is now given by

$$\begin{array}{c} h_{}\left(S\left(t\right),I_{o}\left(t\right),I_{c}\left(t\right)\right)=(\alpha +\beta_{o}\left(t\right)\;{\left[I_{o}\left(t\right)\right]}^{w_{o}}+\beta_{c}\left(t\right)\;{\left[I_{c}\left(t\right)\right]}^{w_{c}})\cdot {\left[S\left(t\right)\right]}^{v} \end{array}$$ (12)

as opposed to *βS*(*t*)*I*(*t*) in (5) for the SEIR model. Fig 2 gives the flow chart of the modified SEIR model and its associated parameters.

A key difference of the model formulation in (6)–(12) is the power transmission dynamics used to model heterogeneity in the population; see [6, 7]. The epidemic models developed in [6, 7] incorporate parametric heterogeneity, that is, these models describe a heterogeneous population where individuals in the population have different trait values from one person to another. In this framework, disease characteristics related to the population, such as the likelihood of a successful transmission for a susceptible or the rate of infectivity of an infectious individual, can be taken to vary heterogeneously. This heterogeneity is characterized via probability distributions, and is known as distributed susceptibility and infectivity in [6, 7]. When a susceptible and infectious trait, respectively, has a *Gamma*(*k*_1_, *ν*_1_) and *Gamma*(*k*_2_, *ν*_2_) distribution (where *Gamma*(*k*, *ν*) refers to the Gamma distribution with shape parameter *k* > 0 and scale parameter *ν* > 0), it is shown in [6, 7] that the resulting epidemic model has power law transmission characteristics with the rate of incidence (of new infections) given by

$$\begin{array}{c} h\left(S\left(t\right),I\left(t\right)\right)\propto {\left[S\left(t\right)\right]}^{q_{1}}\;{\left[I\left(t\right)\right]}^{q_{2}}, \end{array}$$ (13)

where the powers on *S* and *I* are *q*_1_ = 1 + 1/*k*_1_ and *q*_2_ = 1 + 1/*k*_2_ depending on the shape parameter of the corresponding Gamma distribution assumed for distributed susceptibility and infectivity, respectively. Since *k*_1_, *k*_2_ > 0, it follows that *q*_1_, *q*_2_ > 1. The values of *q*_1_ = 1 and *q*_2_ = 1 corresponding to (5) can be thought as the limiting case when *k*_1_ → ∞ and *k*_2_ → ∞ representing homogeneity. From this discussion, it follows that the powers *v*, *w*_*o*_ and *w*_*c*_ on *S*(*t*), *I*_*o*_(*t*) and *I*_*c*_(*t*), respectively, are such that *v* ≥ 1, *w*_*o*_ ≥ 1 and *w*_*c*_ ≥ 1. The lower bounds on *v*, *w*_*o*_ and *w*_*c*_ recover the original SEIR model dynamics with no heterogeneity. The remaining parameters have the following interpretation: (1) *α* represents a small yet significant force of infection that starts the local infection process but is eventually overwhelmed by it. In our context, *α* represents the initial force of infection arising from, say, foreign infectious individuals attending the large gathering at Sri Petaling starting February 27th. (2) The parameters *γ*_*o*_ and *γ*_*c*_ have the same interpretation as *γ* in (3), that is, they are rates of infectiousness but for the observed and asymptomatic compartments, respectively. (3) The parameter *δ* is the same as before, namely, it is the rate of incubation associated with the exposed compartment. (4) The functions *β*_*o*_(*t*) and *β*_*c*_(*t*) are time varying transmission rates for the observed and asymptomatic compartments, respectively, in the modified SEIR model with *β*_*c*_(*t*) = *μβ*_0_(*t*) and *μ* ∈ [0, 1]. In other words, we assume that the transmission rate for asymptomatic individuals is smaller than that of symptomatic individuals; this is a plausible assumption to make as asymptomatic individuals generally possess a lower viral load which leads to lower chances of a successful transmission. On the other hand, a longer asymptomatic infectious period may compensate for the lower transmission rates for an asymptomatic individual, and this possibility is captured by the model via the parameter *γ*_*c*_.

A change-point is incorporated into the modified SEIR model to capture the shift in disease dynamics before and after the start of the MCO. Several works in the COVID literature have incorporated change-points to study the effectiveness of interventions; see, for example, [24–26]. To incorporate our change-point, an unknown threshold date, T*, is chosen so that observed daily cases fall either to the left or right of T*. We denote the observation window to the left of T* by $W_{L}$ consisting of dates from March 1st up to and including T*. The window to the right of T* is denoted by $W_{R}$ which consists of dates after T* up to and including April 28th, 2020. The change-point date T* is inferred from data; it is not taken as March 18th, 2020 which is the date of the start of MCO. Choosing T* in this data-driven way is justified as the impact of MCO on observed cases may not be realized immediately. The modified SEIR model with change-point T* is governed by the ODE system (6)–(12) for all time points $t\in W_{L}$. For the parameters of the modified SEIR model in $W_{L}$, we denote them using the same symbol as before but with a subscript *L*. For $W_{R}$, the same ODE system (6)–(12) is considered but now with a different set of parameter values in $W_{R}$ compared to $W_{L}$ which are denoted using a subscript *R*.

The transmission rates in $W_{L}$ are assumed to be constant, that is, $\beta_{o,L}=\gamma_{o,L}\;e^{a_{0,L}}$ and *β*_*c*,*L*_ = *μ*_*L*_ *β*_*o*,*L*_. The transmission rate for the *I*_*o*_ compartment in $W_{R}$ is taken as

$$\begin{array}{c} \beta_{o,R}\left(t\right)=\gamma_{o,R}[e^{a_{0,R}+a_{1}\;\left(t-T^{*}\right)}+a_{2}\;[1-e^{a_{1}\;\left(t-T^{*}\right)}]], \end{array}$$ (14)

to model possible changes in disease transmission over time. The functional form of *β*_*o*,*R*_(*t*) in (14) has an initial value of $\gamma_{o,R}\;e^{a_{0,R}}$ at *t* = T* after which it decreases (provided *a*_1_ < 0) to the asymptotic value of *a*_2_ *γ*_*o*,*R*_ as *t* → ∞. Thus, *a*_2_ *γ*_*o*,*R*_ represents the residual disease transmission that may be present even during the MCO period, for example, due to close contact between family members in the same household. The general functional form of *β*_*o*,*R*_(*t*) subsumes the constant disease transmission rate model as a special case by taking *a*_1_ = *a*_2_ = 0 in (14). The constant rate submodel has the advantage of not explicitly assuming any functional form for the change in disease transmission over time. On the other hand, it can only ascertain if there is an overall change (increase or decrease) in transmission after the change point T*. Similar to the relationship *β*_*c*,*L*_ = *μ*_*L*_ *β*_*o*,*L*_ in $W_{L}$, we assume *β*_*c*,*R*_(*t*) = *μ*_*R*_ *β*_*o*,*R*_(*t*) for $W_{R}$ for a possibly different *μ* value.

In the subsequent text, several redundancies are removed based on the interpretation of the parameters involved. These simplications are reasonable and lead to a reduction in the number of unknown parameters. This reduction, in turn, improves the model fitting and inference procedures. It is reasonable to assume that *γ*_*c*,*L*_ = *γ*_*c*,*R*_ ≡ *γ*_*c*_, *δ*_*L*_ = *δ*_*R*_ ≡ *δ*, *μ*_*L*_ = *μ*_*R*_ ≡ *μ* and *p*_*L*_ = *p*_*R*_ ≡ *p*; in other words, intervention does not change the values of these parameter in $W_{L}$ and $W_{R}$. These parameters can be assumed constant since they relate to intrinsic characteristics of the disease and of the underlying population. The parameter *γ*_*c*_ relates to asymptomatic infections which are not detected in both $W_{L}$ and $W_{R}$, *δ* relates to the incubation period of the disease, *μ* refers to the transmissibility ratio of asymptomatic to symptomatic individuals (which depends on the viral load of infectees only), and *p* refers to the proportion of symptomatic versus asymptomatic infectees. Hence, these parameters are characteristics of the overall population and the disease, and not the intervention. However, we do not assume that *γ*_*o*,*L*_ = *γ*_*o*,*R*_ for the symptomatic infectious periods, but rather 1/*γ*_*o*,*R*_ ≤ 1/*γ*_*o*,*L*_ since the process of detecting and isolating symptomatic individuals during MCO was generally quicker and more efficient in $W_{R}$ compared to $W_{L}$, resulting in a shorter symptomatic infectious period in $W_{R}$ compared to $W_{L}$.

In the Results section, the constant transmission rate submodel is first fitted to observed data to determine if the MCO is succesful in reducing the overall disease transmission. Only when this is established, the full change-point model, with the explicit decay form of (14) and *a*_1_ < 0, is fitted to the observed data. Further, in the model formulation of (6)–(11), only the *R*_*o*_ compartment is modelled directly using a likelihood function based on daily observed cases. The other compartments of the modified SEIR model remain latent and do not have any direct observation processes for modelling based on likelihoods; see the Bayesian Inference section for further details.

### Quantitative assessment of disease spread

The basic reproduction number, *ℛ*_0_, is defined as the number of secondary infections caused by one primary individual during his/her infectious period. It is the most important quantitative indicator reported to assess whether the disease is in control or not. It is well-known that the threshold value of 1 for *ℛ*_0_ distinguishes between the situations where a major epidemic occurs versus the disease dying out before a major outbreak can become established in the population. In fact, several studies in the literature report a gradual decrease in *ℛ*_0_ after lockdown [27–29]. For the SEIR model in (1)–(4), *ℛ*_0_ is given by the well-known formula *ℛ*_0_ = *β*/*γ* Time-varying measures of secondary infections per primary infected, *ℛ*_*t*_, are also available in the literature. However, for the modified SEIR model, *ℛ*_0_ and *ℛ*_*t*_ cannot be computed. Therefore, we resort to an alternative quantitative assessment of disease spread—the total number of infections (i.e., generational) caused by the introduction of one additional infectious individual into the infection process at time point *t*. This procedure is illustrated using the *I*_*o*_ compartment. Based on (6)–(12), new values for the ODE system are calculated from time point *t* onwards with current state values serving as initializations of the ODE system for all except one compartment: For the *I*_*o*_-compartment, the current value *I*_*o*_(*t*) is replaced by *I*_*o*_(*t*) + 1/*N* as the initial value. The new rate of incidence is given by $h(S^{*},I_{o}^{*},I_{c}^{*})$ over the infectious period of the individual when the ODE system is propagated using (6)–(12) in [*t*, *t* + 1/*γ*_*o*_]. The increase in the rate of incidence by the introduction of this individual in the *I*_*o*_ compartment is given by

$$\begin{array}{c} \Delta_{t}\left(I_{o}\right)=\int_{\left[t,t+\frac{1}{\gamma_{o}}\right]}\;[h(S^{*}\left(u\right),I_{o}^{*}\left(u\right),I_{c}^{*}\left(u\right))-h(S\left(u\right),I_{o}\left(u\right),I_{c}\left(u\right))]\;du. \end{array}$$ (15)

Similarly, the increase in the rate of incidence by the introduction of one infectious individual into the *I*_*c*_ compartment at time point *t* can be calculated. This is denoted by Δ_*t*_(*I*_*c*_). The final increase in the incidence is the mixture

$$\begin{array}{c} \Delta_{t}=p\;\Delta_{t}\left(I_{o}\right)+\left(1-p\right)\Delta_{t}\left(I_{c}\right) \end{array}$$ (16)

where *p* is the proportion of exposed individuals who enter the *I*_*o*_ compartment in (8).

### Bayesian inference

Model fitting and inference is carried out in a Bayesian framework.

#### The likelihood

The total numbers of daily cases, $D_{t},\;t\in W_{L}\cup W_{R}$, which constitute noisy observations from the *R*_*o*_-compartment in the modified SEIR model, are assumed to be distributed as

$$\begin{array}{c} D_{t}\overset{\text{iid}}{∼}NB\left(\cdot \;;\;\gamma_{o}\;I_{o}\left(t\right),\;\tau \right) \end{array}$$ (17)

where *NB*(*x*;*ν*, *τ*) denotes the negative binomial probability function with mean *ν* and variance *ν* + *τν*^2^. The parameter *τ* measures overdispersion with respect to the Poisson probability function which is recovered from *NB*(*x*;*ν*, *τ*) when *τ* = 0; see, for example, [30, 31]. The observed data on daily cases in Malaysia exhibited significant overdispersion, and as a result, the Poisson likelihood did not fit the observed cases well. More discussion on this aspect is presented later.

#### Assignment of priors

Several parameters of the modified SEIR model in (6)–(12) take different values in $W_{L}$ and $W_{R}$, and reflect changes in the disease trajectory due to intervention. Other parameters, including those that relate to the intrinsic characteristics of the disease, remain constant throughout $W_{L}$ and $W_{R}$. Defining

$$\Theta_{H}\equiv \{\;a_{0,H},\;\gamma_{o,H},\;w_{o,H},\;w_{c,H},\;v_{H},\;\alpha_{H}\;\}$$

for *H* ∈ {*L*, *R*}, we denote the collection of all parameters of the full change-point model in $W_{L}\cup W_{R}$ by

$$\Theta \equiv \Theta_{L}\cup \Theta_{R}\cup \{\;\delta ,\;\gamma_{c},\;p,\;\tau ,\;T^{*},\;a_{1},\;a_{2},\;e_{0},\;i_{0}\;\},$$

where all notation used for the parameters is as before. In the subsequent discussion, separate priors are elicited either on individual components of Θ, or on specific subsets of Θ where the parameters within these subsets satisfy certain restrictions. A full prior on Θ then follows from assuming independence between the separate prior assignments. For each *ξ*_1_ ∈ {*p*, *μ*}, the prior distribution on *ξ*_1_ is assumed to be uniform, $U(A_{\xi_{1}},B_{\xi_{1}})$ with hyperparameters $A_{\xi_{1}}$ and $B_{\xi_{1}}$, representing the lower and upper bounds, respectively. Since each *ξ*_1_ represents a proportion, we have $A_{\xi_{1}}\geq 0$ and $B_{\xi_{1}}\leq 1$; however, the choices of hyperparameters used in the experiments for all parameters, including *ξ*_1_ ∈ {*p*, *μ*}, will be discussed later. For now, we provide only the explicit forms of the priors assumed on the components of Θ.

The parameters that relate to the infectious periods, *γ*_*o*,*L*_, *γ*_*o*,*R*_ and *γ*_*c*_, satisfy the relations 1/*γ*_*o*,*R*_ ≤ 1/*γ*_*o*,*L*_ (as pointed out earlier) and 1/*γ*_*o*,*L*_ < 1/*γ*_*c*_. The latter condition follows from the fact that the asymptomatic infectious period, being undetected, will generally be longer than its symptomatic counterpart in $W_{L}$, since symptomatic individuals will be isolated once they test positive. The joint prior distribution on {*γ*_*o*,*L*_, *γ*_*o*,*R*_, *γ*_*c*_} can be specified according to the following generation scheme: We first generate $1/\gamma_{o,R}∼U\left(A_{\gamma_{o,R}},B_{\gamma_{o,R}}\right)$, then set 1/*γ*_*o*,*L*_ = 1/*γ*_*o*,*R*_ + *ξ*_2_, and finally take 1/*γ*_*c*_ = 1/*γ*_*o*,*L*_ + *ξ*_3_ where $\xi_{j}∼U\left(A_{\xi_{j}},B_{\xi_{j}}\right)$ with $A_{\xi_{j}}=0$ and $B_{\xi_{j}}>0$, independently for *j* = 2 and 3. We refer to the infectious periods reported in literature, namely [18–20, 32], to choose reasonable values for $A_{\gamma_{o,L}}$, $B_{\gamma_{o,L}}$, $B_{\xi_{2}}$ and $B_{\xi_{3}}$. Based on the reported values, infectious periods within 2–8 days are found to provide a reasonable range for our experiments. The prior on *δ*, where 1/*δ* is the incubation period, is taken as 1/*δ* ∼ *U*(*A*_*δ*_, *B*_*δ*_). To select reasonable values for the lower and upper bounds, we refer to the reported literature (for example, see [33]) and consider an incubation period between 2–13 days to be a satisfactory range for our study.

The proposed Bayesian methodology calls for Θ-samples to be generated from its full prior specification as described above. For this purpose, explicit mathematical experssions of the joint prior density function on parameter subsets of Θ, such as {*γ*_*o*,*L*_, *γ*_*o*,*R*_, *γ*_*c*_}, are not required. One only needs to be able to simulate the reciprocals based on the generation scheme described above, and then invert the reciprocals to obtain prior samples of *γ*_*o*,*L*_, *γ*_*o*,*R*_ and *γ*_*c*_ from their implicit joint density specification.

Next, the prior on *τ* is taken as *τ* ∼ *U*(*A*_*τ*_, *B*_*τ*_) where 0 ≤ *A*_*τ*_ < *B*_*τ*_, and *B*_*τ*_ is a pre-specified positive constant representing the maximum extent of overdispersion apriori. The prior on *α*_*L*_ is assumed to be $U(A_{\alpha_{L}},B_{\alpha_{L}})$, where $A_{\alpha_{L}0}=0$ and $B_{\alpha_{L}}>0$ represents the maximum value of a small but constant force of infection arising, for example, from infectious foreign attendees of the Sri Petaling gathering. The parameter *α*_*R*_ is set to 0 as there is no such force of infection in $W_{R}$. For the parameters *w*_*o*,*L*_, *w*_*o*,*R*_ and *v*_*L*_, independent uniform priors are considered with lower and upper bounds given by $A_{w_{o,L}}$ and $B_{w_{o,L}}$ for *w*_*o*,*L*_, $A_{w_{o,R}}$ and $B_{w_{o,R}}$ for *w*_*o*,*R*_, and $A_{v_{L}}$ and $B_{v_{L}}$ for *v*_*L*_, respectively. Since these parameters have a natural lower bound of 1 that represents homogeneity, the hyperparameters of the uniform priors are chosen in the range [1, ∞). The priors on *w*_*c*,*L*_, *w*_*c*,*R*_ and *v*_*R*_ are taken using the following relations: *w*_*c*,*L*_ = *w*_*o*,*L*_ + *ξ*_4_, *w*_*c*,*R*_ = *w*_*o*,*R*_ + *ξ*_5_, and *v*_*R*_ = *v*_*L*_ + *ξ*_6_ where $\xi_{j}∼U\left(A_{\xi_{j}},B_{\xi_{j}}\right)$ with $A_{\xi_{j}}=0$ and $B_{\xi_{j}}>0$, independently of each other for *j* = 4, 5 and 6.

The prior on the change-point T* is taken to be uniform on dates from March 18th, 2020, to March 31st, 2020, both inclusive. Since we define T* as the number of days after March 1st 2020, T* ∼ *U*(17, 30) a priori.

The constant contact rate *β*_*o*,*L*_ during the pre-MCO period cannot be determined by direct observation. Hence, putting a prior directly on *β*_*o*,*L*_ is difficult. However, since $\beta_{o,L}=e^{a_{0,L}}\;\gamma_{o,L}$, the quantity $e^{a_{0,L}}$ has the interpretation of a reproduction number, a more fundamental quantity for infectious diseases compared to *β*_*o*,*L*_. Thus, one can obtain a range of values for *a*_0,*L*_ from previous reports on similar flu-like epidemics (see, for example, [34]) where we found the range 0–3.5 appropriate for covering the true value of *a*_0,*L*_ in our case. We take the prior on *a*_0,*L*_ as $a_{0,L}∼U\left(A_{a_{0,L}},B_{a_{0,L}}\right)$ where $A_{a_{0,L}}\geq 0$ and $B_{a_{0,L}}\leq 3.5$ based on benchmark values in [34]. The function *β*_*o*,*R*_(*t*) has the form in (14) which depends on coefficients *a*_0,*R*_, *a*_1_ and *a*_2_. For the prior on *a*_0,*R*_, we consider two cases: When using the constant rate submodel to determine if an overall reduction in transmission has occurred or not, the prior on *a*_0,*R*_ is chosen as $a_{0,R}∼U\left(A_{a_{0,R}},B_{a_{0,R}}\right)$ with $A_{a_{0,R}}<0$ and $B_{a_{0,R}}>0$. This ensures that the prior support includes both positive and negative values to represent possible increase and decrease in the transmission rate. On the other hand, if the full model of (14) is considered, *a*_0,*R*_ is chosen deterministically (hence, no prior) to ensure continuity of the infection process before and after T*. Specifically, *a*_0,*R*_ is chosen so that the incidence rate of new infections, *h*(*S*(*t*), *I*_*o*_(*t*), *I*_*c*_(*t*)), in $W_{L}$ and $W_{R}$ coincide at *t* = T*. In the case of the full change-point model, *a*_1_ is given a uniform prior with support on $\left[A_{a_{1}},B_{a_{1}}\right]$. We take $A_{a_{1}}<0$ and $B_{a_{1}}=0$ if a reduction in disease transmission is first established by the constant rate submodel. For the prior on *a*_2_, note that $0\leq a_{2}\leq e^{a_{0,R}}$. Hence, a reasonable prior to put on *a*_2_ is $U(0,e^{a_{0,R}})$.

Next, we describe the prior assignment on the initial number of symptomatic infectious and exposed individuals, denoted by *i*_0_ and *e*_0_, respectively. Given *δ* and *γ*_*o*,*L*_, whose priors were elicited earlier, the prior on *i*_0_ is taken as $i_{0}∼U\left(m_{i_{0}}-\Delta_{i_{0}},m_{i_{0}}+\Delta_{i_{0}}\right)$, where the midpoint $m_{i_{0}}$ and half-wdith $\Delta_{i_{0}}$ depend on *δ* and *γ*_*o*,*L*_. Reasonable choices of the midpoint $m_{i_{0}}$ and half-width $\Delta_{i_{0}}$ are made based on the differential equation for the *R*_*o*_ compartment. Noting that ${\dot{R}}_{o}\left(t\right)=\gamma_{o,L}I_{o}\left(t\right)$ for $t\in W_{L}$ from (9), we get $i_{0}={\dot{R}}_{o}\left(0\right)/\gamma_{o,L}$. To obtain an estimate of ${\dot{R}}_{o}\left(0\right)$, a second-order polynomial is fitted using least squares to the trajectory of cumulative cases in a window of *m* ≥ 3 days starting from day 0. ${\dot{R}}_{o}\left(0\right)$ is then estimated by taking the first derivative of the fitted polynomial and evaluating it at time *t* = 0. Subsequently, $m_{i_{0}}$ is set as ${\dot{R}}_{o}\left(0\right)/\gamma_{o,L}$. The half-width $\Delta_{i_{0}}>0$ is fixed to be reasonably large to represent the maximum extent of prior uncertainty around $m_{i_{0}}$. Similar to *i*_0_, the prior on the initial number of exposed individuals, *e*_0_, is taken as $e_{0}∼U\left(m_{e_{0}}-\Delta_{e_{0}},m_{e_{0}}+\Delta_{e_{0}}\right)$ for the midpoint $m_{e_{0}}$ and half-width $\Delta_{e_{0}}$. The midpoint $m_{e_{0}}$ is determined similarly as before using the value of the second derivative of the fitted polynomial evaluated at time *t* = 0 and the generated value of *δ*. The half-width $\Delta_{e_{0}}>0$ is once again fixed to be large to represent the maximum extent of prior uncertainty around $m_{e_{0}}$.

The model fitting experiments were conducted either by choosing default values for the lower and upper bounds, or by choosing values guided by literature when such information is available. For all other hyperparameters where no information is available, reasonable values were chosen at the start of the experiments, and the hyperparameters were adjusted by repeated trial and error runs to achieve the best fit models to the observed data akin to the empirical Bayes approach [35], at least informally. Table 1 gives the values of the hyperparameters used to obtain the plots and inference in the Results section.

**Table 1:** Hyperparameter values for the reported results.

	Malaysia		Selangor		Sarawak	
Parameter	*A*	*B*	*A*	*B*	*A*	*B*
*p*	0.5	1.0	0.5	1.0	0.5	1.0
*μ*	0.0	1.0	0.0	1.0	0.0	1.0
1/*γ*_*o*,*R*_	6.0	7.5	6.0	7.5	6.0	8.0
*ξ*_2_	0.0	1.0	0.0	1.0	0.0	1.0
*ξ*_3_	0.0	1.0	0.0	1.0	0.0	1.0
1/*δ*	6.0	8.0	5.5	8.0	7.0	8.0
*τ*	0.1	0.25	0.1	0.6	0.0	1.0
*α*_*L*_	0.0	10^−6^	0.0	10^−6^	0.0	10^−6^
*w*_*o*,*L*_	1.0	1.15	1.0	1.15	1.0	1.10
*ξ*_4_	0.0	6.0	0.0	6.0	0.0	6.0
*w*_*o*,*R*_	1.0	1.5	1.0	2.0	1.0	1.1
*ξ*_5_	0.0	6.0	0.0	6.0	0.0	6.0
*v*_*L*_	1.0	6.0	1.0	6.0	1.0	6.0
*ξ*_6_	0.0	6.0	0.0	6.0	0.0	6.0
T*	17	30	17	30	17	30
*a*_0,*L*_	1.5	3.5	1.0	3.0	0.0	3.0
*a*_0,*R*_	−3	3	−3	3	−3	3
*a*_1_	−2.5	0	−3.0	0	−2.0	0

• The upper and lower bounds of *a*_2_, *i*_0_ and *e*_0_ are, respectively, 0 and $e^{a_{0,R}}$ for *a*_2_, $m_{i_{0}}+\Delta_{i_{0}}$ and $m_{i_{0}}-\Delta_{i_{0}}$ for *i*_0_, and $m_{e_{0}}+\Delta_{e_{0}}$ and $m_{e_{0}}-\Delta_{e_{0}}$ for *e*_0_. These upper and lower bounds are functions of the parameters given in the above table.

• The upper and lower bounds reported for *a*_0,*R*_ are for the constant rate submodel.

#### Computational algorithm

Based on the negative binomial likelihood and prior elicitation in The Likelihood and Assignment of Priors, respectively, the posterior of Θ can be derived using Bayes theorem as

$$\pi (\Theta \;|\;D)\propto \pi \left(\Theta \right)\;\prod_{t\in W_{L}}\;NB\left(D_{t}\;;\;\gamma_{o,L}\;I_{o}\left(t\right),\;\tau \right)\prod_{t\in W_{R}}\;NB\left(D_{t}\;;\;\gamma_{o,R}\;I_{o}\left(t\right),\;\tau \right)$$ (18)

=π(Θ)·L(Θ) (19)

where $D=\{D_{t}\;:\;t\in W_{L}\cup W_{R}\}$ is the collection of observed daily cases from March 1st until April 28, 2020, **L**(Θ) is the entire likelihood for **D** and *π*(Θ) is the prior on Θ as described in Assignment of Priors. Bayesian inference is carried out using Monte Carlo importance sampling. A total of *M* samples, Θ_*i*_ for *i* = 1, 2, ⋯, *M*, are generated from the prior specification *π*(Θ). The likelihood, **L**(Θ_*i*_), is computed for each Θ_*i*_ and normalized to obtain weights

$$\begin{array}{c} w_{i}=\frac{L(\Theta_{i})}{\sum_{i=1}^{M}\;L\left(\Theta_{i}\right)} \end{array}$$ (20)

for *i* = 1, 2, ⋯, *M*. The ensemble of weights and samples ${\left\{w_{i},\Theta_{i}\right\}}_{i=1}^{M}$ constitute a weighted sample from the posterior distribution *π*(Θ|**D**) and any summary posterior measure can be obtained from the ensemble via Monte Carlo. For any function *g* of Θ, the posterior expectation of *g*(Θ),

$$\begin{array}{c} E\left(g\left(\Theta \right)\right)\equiv \int_{\Theta }\;g\left(\Theta \right)\;\pi \left(\Theta \;|\;D\right)\;d\Theta , \end{array}$$

is approximated by the corresponding ensemble average

$$\begin{array}{c} {\hat{g}}_{M}\equiv \frac{1}{M}\sum_{i=1}^{M}\;w_{i}\;g\left(\Theta_{i}\right), \end{array}$$

the latter being an unbiased estimator of *E*(*g*(Θ)) with variance proportional to 1/*M* which tends to 0 as *M* → ∞. Thus, ${\hat{g}}_{M}$ becomes a more accurate estimate of *E*(*g*(Θ)) as *M* → ∞. Different choices of *g* are used to obtain the posterior means, variances and credible intervals of parameters in Θ.

Resampling from the ensemble ${\left\{w_{i},\Theta_{i}\right\}}_{i=1}^{M}$ converts it to a new ensemble of weights and samples ${\left\{w_{i}^{*},\Theta_{i}^{*}\right\}}_{i=1}^{M}$ where the new weights are now uniform, i.e., $w_{i}^{*}=1/M$. The usual sample mean and sample variance of ${\left\{\Theta_{i}^{*}\right\}}_{i=1}^{M}$ can now be used to provide an (unbiased) estimate of *E*(*g*(Θ)) and its corresponding variance. The above importance sampling methodology is easy to code and implement but one has to be cautious so that the distribution of weights is not too extreme. If the prior on Θ is chosen such that it spans a considerably larger region compared to where the likelihood concentrates its mass, the distribution of weights will be become extreme. As a result, almost all of the weights ${\left\{w_{i}\right\}}_{i=1}^{M}$ will be extremely small (close to zero) and only a few will have large positive values. Resampling these weights will select Θ_*i*_-samples associated to these large weights multiple times and this will degrade the estimation of *E*(*g*(Θ)). Thus, it is important to judiciously choose the prior ranges, where possible, so that it adequately covers regions of high likelihood values. This is achieved in the Results section by running different selections of the hyperparameters and judiciously adjusting the range of the priors (where possible and respecting the information on the disease and intervention dynamics) to obtain the best fitting models. Based on this importance sampling method as well, an approximation to the Maximum-a-Posteriori (MAP) estimator of Θ can be found since

$$\begin{array}{c} {\hat{\Theta }}_{MAP}\equiv \underset{\Theta }{\text{arg}\;\text{max}}\;\pi \left(\Theta \;|\;D\right)\approx \underset{1\leq i\leq M}{\text{arg}\;\text{max}}\;[w_{i}\;\pi \left(\Theta_{i}\right)]\equiv {\hat{\Theta }}_{M}, \end{array}$$ (21)

say, for large *M*. To see why the above approximation is valid, we partition Θ into Θ = (Ψ, T*) where Ψ consists of all continuous parameters in Θ and T* is the change point which takes on finitely many values apriori. Correspondingly, partition ${\hat{\Theta }}_{MAP}$ as ${\hat{\Theta }}_{MAP}=\left(\Psi_{MAP}^{},T_{MAP}^{*}\right)$. Further assume that the posterior $\pi (\left(\Psi ,T_{MAP}^{*}\right)\;|\;D)$ is continuous at Ψ = Ψ_*MAP*_ and ${\hat{\Theta }}_{MAP}$ lies in the interior of *supp*[*π*(Θ|**D**)] where

supp[π(Θ|D)]={Θ:π(Θ|D)>0}.

The occurence of the event $E_{M}\equiv \left\{\;\right|{\hat{\Theta }}_{MAP}-{\hat{\Theta }}_{M}\left|>\epsilon \;\right\}$ for an arbitrarily small *ϵ* > 0 implies that there exists a neighbourhood $N\equiv (N_{0},T_{MAP}^{*})$ of ${\hat{\Theta }}_{MAP}$ which is contained in *supp*[*π*(Θ|**D**)] with $\lambda \equiv \int_{N_{0}}\pi \left(\left(\Psi ,T_{MAP}^{*}\right)|D\right)\;d\Psi >0$, and none of the *M* samples ${\left\{\Theta_{i}\right\}}_{i=1}^{M}$ lie in N. Hence,

$$\begin{array}{cc} P(|{\hat{\Theta }}_{MAP}-{\hat{\Theta }}_{M}|>\epsilon )=P(E_{M}) & \leq P(\Theta_{i}\notin N\;\text{for}\;\text{all}\;i=1,2,\cdots ,M)\\ & ={\left(1-\lambda \right)}^{M}\to 0 \end{array}$$

as *M* → ∞. In other words, as *M* becomes large, the sampling based maximum ${\hat{\Theta }}_{M}$ will be close to the true MAP estimate ${\hat{\Theta }}_{MAP}$ with probability tending to 1. The approximation in (21) could fail if any one of the assumptions made above is not valid: The posterior $\pi (\left(\Psi ,T_{MAP}^{*}\right)|D)$ could be discontinuous at Ψ = Ψ_*MAP*_ or ${\hat{\Theta }}_{MAP}$ may not lie in the interior of *supp*[*π*(Θ|**D**)]. However, discontinuity is not a concern here as $\pi (\left(\Psi ,T_{MAP}^{*}\right)\;|\;D)$ is a continuous (in fact, differentiable) function of Ψ. To ensure that ${\hat{\Theta }}_{MAP}$ lies in the interior of *supp*[*π*(Θ|**D**)], the experiments were run based on judicious choices of the prior hyperparameters which has been mentioned earlier as well. The resampled ${\left\{\Theta_{i}^{*}\right\}}_{i=1}^{M}$ values are used for plotting and providing a quantification of uncertainty around ${\hat{\Theta }}_{MAP}$ (see, for example, Figs 10 and 11 in the Results section). Numerical measures of uncertainty can be obtained, for example, by computing the usual sample variance based on ${\left\{g\left(\Theta_{i}^{*}\right)\right\}}_{i=1}^{M}$ to describe the variablity around the sample mean which in this case is an unbiased estimate of *E*(*g*(Θ)).

The Bayesian computational algorithm described in this section is developed using R and RStudio^®^. The likelihood **L**(Θ) for a specific Θ is evaluated numerically from the solution of the ODE system (6)–(12) obtained using the deSolve package in R.

## Results

The study period is from March 1, 2020 until April 28, 2020 with *T*_0_ = 0 and *T*_1_ = 59. The impacts of the Sri Petaling gathering and MCO implementation are analyzed here using the proposed model described in The modified SEIR model section. Reported daily cases at the national level as well as for Selangor and Sarawak are used for model fitting and parameter estimation. Note that all states in Malaysia implemented MCO Phases 1–3 using the same guidelines and protocols. Thus, one can gauge the impact of the Sri Petaling gathering on COVID-19 spread in Malaysia based on a comparison between states and the national experience. Here, Selangor and Sarawak are chosen as two such representative states with high and low population densities, respectively.

**Fig 3:**
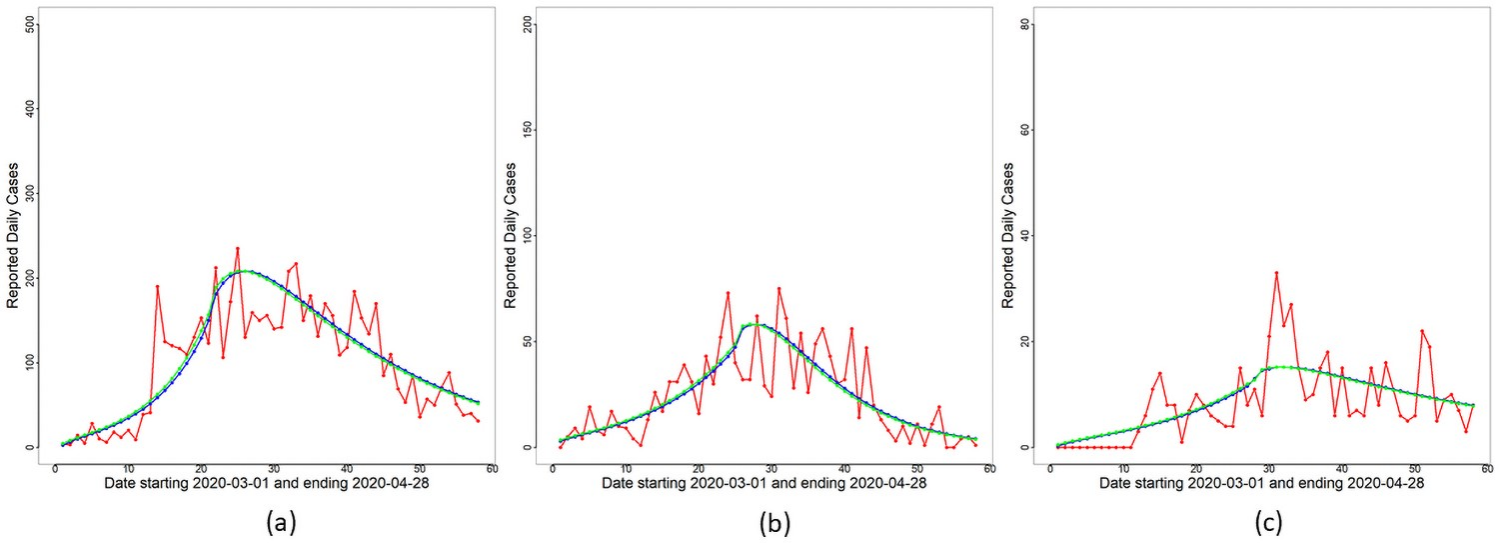
Constant rate submodel—Daily cases. Reported daily cases (red line), and overlay plots of Δ*R*_*o*_(*t*) (blue line) and *F*(*t*) (green line) based on ${\hat{\Theta }}_{MAP}$ for the constant rate submodel for (a) Malaysia, (b) Selangor and (c) Sarawak.

**Fig 4:**
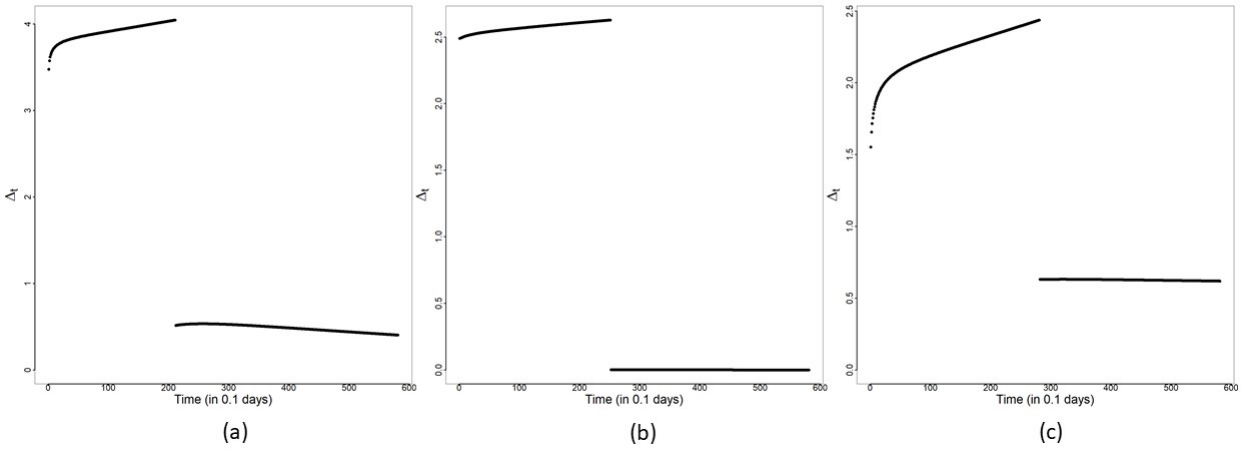
Constant rate submodel—Δ_*t*_. Plots of Δ_*t*_ versus *t* for the constant rate submodel for (a) Malaysia, (b) Selangor and (c) Sarawak.

First, we investigate if the MCO implementation had an overall effect of reducing COVID-19 transmission rates. For this purpose, the constant rate submodel of (14) is used and the prior on *a*_0,*R*_ in $W_{R}$ is chosen to be uniform with support on both positive and negative values. The Bayesian inference methodology described in the Bayesian Inference section is carried out with *M* = 50, 000 to obtain ${\hat{\Theta }}_{MAP}$ and samples $\Theta_{i}^{*}$ from the posterior of Θ in (19). The curves of *F*(*t*), defined as

$$\begin{array}{c} F\left(t\right)=\{\begin{array}{cc} \gamma_{o,L}\;I_{o}\left(t\right) & \text{if}\;\;\;t\leq T^{*}\\ \gamma_{o,R}\;I_{o}\left(t\right) & \text{if}\;\;\;t>T^{*} \end{array} \end{array}$$

and Δ*R*_*o*_(*t*)≡*R*_*o*_(*t*) − *R*_*o*_(*t* − 1) for each day *t* are obtained based on ${\hat{\Theta }}_{MAP}$ and are displayed in Fig 3. This submodel captures broad features (increasing and decreasing trends) of the reported cases trajectories in all three panels for Malaysia, Selangor and Sarawak. To quantify the overall change in transmission before and after MCO implementation, Δ_*t*_ (see (16)) is obtained for *t* in $W_{L}$ and $W_{R}$. The plots of Δ_*t*_ versus *t* are shown in Fig 4. A consistent feature of the plots in all three panels is that they first increase for time points *t* ≤ T* followed by a significant drop for *t* > T*. Hence, we conclude that an exponential rise in cases occurred right after the completion of the Sri Petaling gathering on March 1st 2020, and the implementation of the MCO successfully stemmed the exponential rise at the national, Selangor and Sarawak levels.

**Fig 5:**
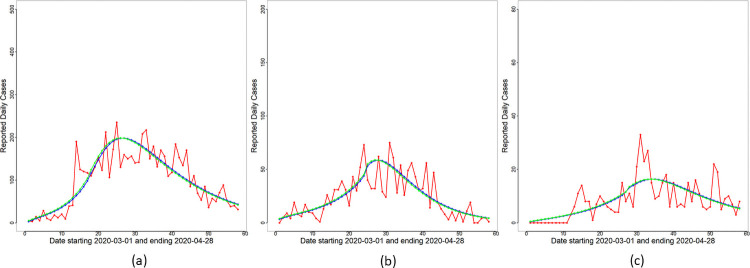
Modified SEIR model: Daily cases. Reported daily cases (red line), and overlay plots of Δ*R*_*o*_(*t*) (blue line) and *F*(*t*) (green line) based on ${\hat{\Theta }}_{MAP}$ for (a) Malaysia, (b) Selangor and (c) Sarawak.

**Fig 6:**
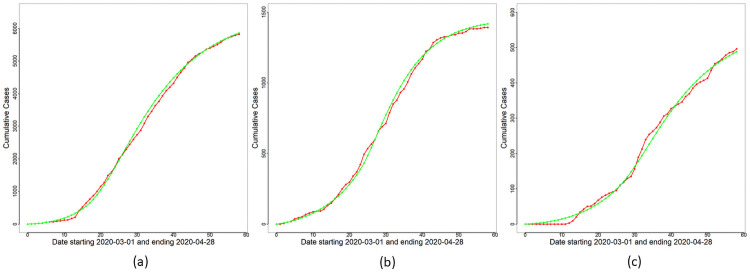
Modified SEIR model: Cumulative cases. Reported cumulative cases (red line), and overlay plots of *R*_*o*_(*t*) (green line) based on ${\hat{\Theta }}_{MAP}$ for (a) Malaysia, (b) Selangor and (c) Sarawak.

With reduced disease transmission established in $W_{R}$, we next proceed to utilize the functional form (14) of *β*_*o*_(*t*) as a quantitative model for transmission decay in $W_{R}$. The Bayesian inference methodology in the Bayesian Inference section is applied to the full model with *M* = 50, 000 to obtain ${\hat{\Theta }}_{MAP}$ and samples $\Theta_{i}^{*}$ from the posterior of Θ in (19). The curves of *F*(*t*) and Δ*R*_*o*_(*t*)≡*R*_*o*_(*t*) − *R*_*o*_(*t* − 1) for each day *t* are obtained based on ${\hat{\Theta }}_{MAP}$ and are displayed in Fig 5. Daily cumulative cases and the curve of *R*_*o*_(*t*) are displayed in Fig 6. We note from these figures that the proposed model captures broad features of the observed data and is an improvement over the constant rate submodel. Uncertainty estimates are obtained for all unknown parameters in Θ based on the ensemble ${\left\{\Theta_{i}^{*}\right\}}_{i=1}^{M}$. Variability estimates can be obtained for all parameters and their functions. As an illustration, we demonstrate the extent of variability inherent in the posterior visually for the *F*(*t*) curve (see (17)) for *t* ∈ [*T*_0_, *T*_1_]. This is displayed in Fig 10 which shows that most of the reported case numbers are well within the limits of variability of the posterior of *F*(*t*). Hence, the proposed model together with the negative binomial likelihood are able to explain the variability in the reported case numbers. However, there are a few exceptions, the most notable being the reported case number on Day 14 for Malaysia in Fig 10(a). We present an explanation for this outlying case later in the section Discussion on Reporting Delays, Case Redistribution and Overdispersed Likelihoods.

**Fig 7:**
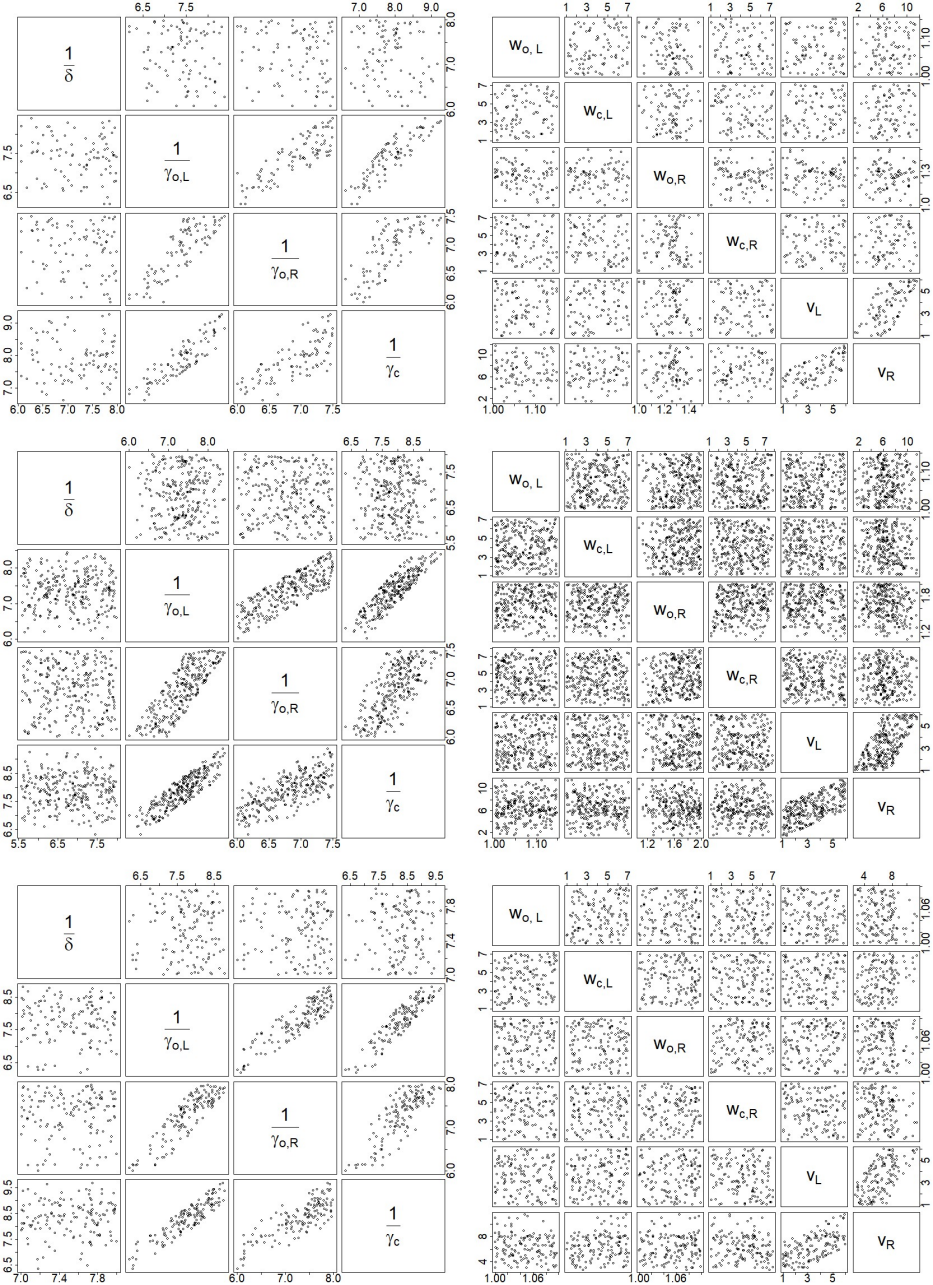
Correlation plots 1. Correlation plots of selected pairs of parameters of the modified SEIR model for Malaysia (top row), Selangor (middle row) and Sarawak (bottom row).

**Fig 8:**
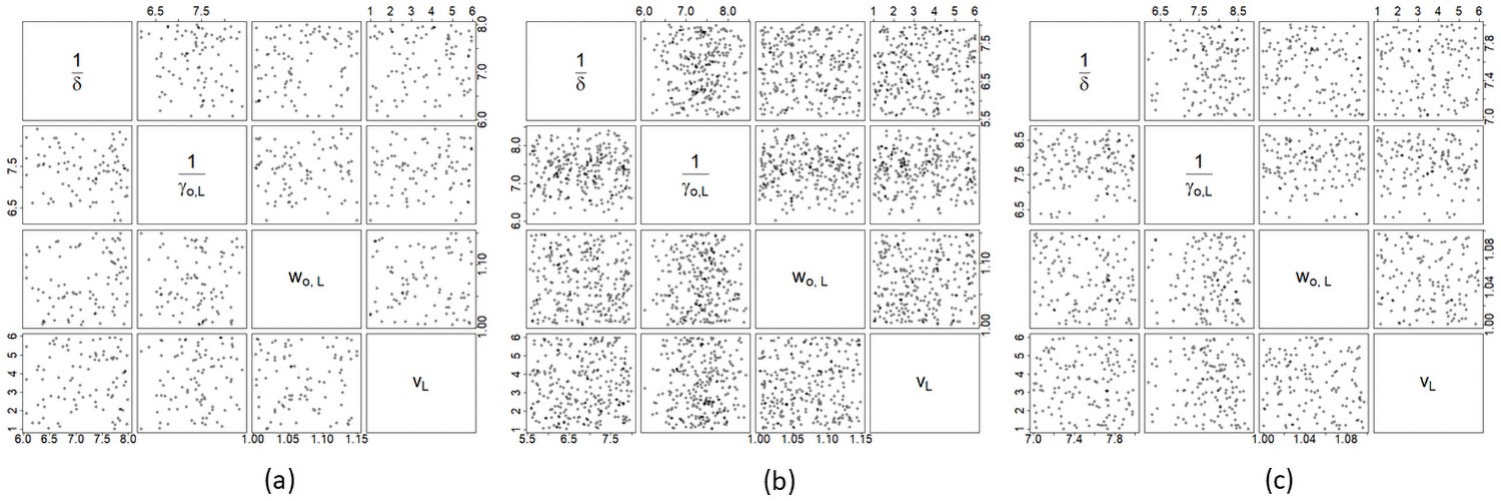
Correlation plots 2. Correlation plots of a different set of pairings of parameters of the modified SEIR model for (a) Malaysia, (b) Selangor and (c) Sarawak.

Further results from the Bayesian analyses are summarized in Tables 2–4. These tables give the MAP estimates of parameters and their corresponding 95% credible intervals for Malaysia, Selangor and Sarawak, respectively. The correlation plots of selected pairs of parameters are provided in Figs 7 and 8. From these figures and others which we did not provide due to space limitations, we find that the posterior samples of parameter pairs are not strongly correlated with each other, except for the pairs for which correlation was induced apriori via the joint prior elicitation.

**Table 2:** MAP estimates and associated credible intervals for Malaysia.

Window	Parameter	MAP	95% Credible Interval
$W_{L}$	*a*_0_	2.458	(1.663, 3.424)
	*w*_*o*_	1.070	(1.008, 1.141)
	*w*_*c*_	1.846	(1.377, 6.795)
	*v*	5.816	(1.313, 5.838)
	*α*	4.69 × 10^−7^	(2.57 × 10^−8^, 9.22 × 10^−7^)
	1/*γ*_*o*_	7.161	(6.604, 8.326)
$W_{R}$	*a*_1_	-0.371	(-2.420, -0.216)
	*a*_2_	4.162	(0.867, 21.381)
	*w*_*o*_	1.221	(1.042, 1.420)
	*w*_*c*_	5.959	(1.295, 7.251)
	*v*	10.345	(3.325, 11.086)
	1/*γ*_*o*_	6.866	(6.194, 7.478)
–	1/*γ*_*c*_	7.661	(7.113, 9.272)
	1/*δ*	7.864	(6.389, 7.940)
	*μ*	0.780	(0.117, 0.932)
	*p*	0.765	(0.518, 0.988)
	*τ*	0.153	(0.127, 0.241)
	*i*_0_	5.786	(1.120, 9.439)
	*e*_0_	255.20	(211.60, 301.88)
	T*	17	(17, 23)

**Table 3:** MAP estimates and associated credible intervals for Selangor.

Window	Parameter	MAP	95% Credible Interval
$W_{L}$	*a*_0_	2.819	(1.121, 2.866)
	*w*_*o*_	1.139	(1.005, 1.146)
	*w*_*c*_	6.113	(1.293, 7.033)
	*v*	4.244	(1.124, 5.887)
	*α*	3.30 × 10^−7^	(7.16 × 10^−9^, 9.68 × 10^−7^)
	1/*γ*_*o*_	7.900	(6.460, 8.327)
$W_{R}$	*a*_1_	-1.756	(-2.830, -0.307)
	*a*_2_	1.830	(0.056, 15.621)
	*w*_*o*_	1.942	(1.220, 1.980)
	*w*_*c*_	3.351	(1.535, 7.464)
	*v*	4.984	(2.053, 10.683)
	1/*γ*_*o*_	6.978	(6.085, 7.470)
–	1/*γ*_*c*_	8.640	(6.808, 9.107)
	1/*δ*	7.190	(5.654, 7.904)
	*μ*	0.881	(0.036, 0.969)
	*p*	0.713	(0.533, 0.986)
	*τ*	0.351	(0.211, 0.542)
	*i*_0_	20.38	(10.52, 30.58)
	*e*_0_	103.86	(67.72, 113.72)
	T*	24	(20, 28)

**Table 4:** MAP estimates and associated credible intervals for Sarawak.

Window	Parameter	MAP	95% Credible Interval
$W_{L}$	*a*_0_	1.605	(0.953, 2.401)
	*w*_*o*_	1.051	(1.002, 1.093)
	*w*_*c*_	6.848	(1.622, 6.879)
	*v*	5.502	(1.112, 5.956)
	*α*	9.18 × 10^−7^	(9, 22 × 10^−9^, 9.74 × 10^−7^)
	1/*γ*_*o*_	7.721	(6.404, 8.754)
$W_{R}$	*a*_1_	-0.325	(-1.908, -0.131)
	*a*_2_	0.573	(0.204, 3.114)
	*w*_*o*_	1.046	(1.006, 1.097)
	*w*_*c*_	2.633	(1.264, 6.603)
	*v*	5.879	(3.410, 11.490)
	1/*γ*_*o*_	7.015	(6.144, 7.942)
–	1/*γ*_*c*_	8.242	(7.036, 9.674)
	1/*δ*	7.433	(7.069, 7.974)
	*μ*	0.784	(0.074, 0.935)
	*p*	0.810	(0.511, 0.966)
	*τ*	0.240	(0.153, 0.585)
	*i*_0_	1.783	(1.217, 8.695)
	*e*_0_	18.06	(15.23, 36.13)
	T*	27	(19, 33)

We provide a summary of the salient findings of our inference procedure. The symptomatic and asymptomatic infectious periods as well as the incubation periods are found to be around 6–8 days for Malaysia, Selangor and Sarawak. These findings are similar to values reported in the literature for other countries; see, for example, [18–20, 32]. Change-points T* are estimated not too far away from the date of MCO implementation, March 18th 2020. For Malaysia, T* = 17 is the MAP estimate which corresponds to March 18th, 2020, and the associated 95% credible interval is (17, 23). For Selangor, the MAP estimate of T* is T* = 24 (March 25th 2020) with (20, 28) being the 95% credible interval. For Sarawak, the transition date is less precise. The MAP estimate is T* = 28 (March 29th 2020) but the 95% credible interval (19, 33) is much larger indicating higher uncertainty in T*. This can be attributed to the fact that the trajectory of reported case numbers for Sarawak shows a slower and more gradual increase, then decrease, compared to Malaysia and Selangor (see Fig 5).

**Fig 9:**
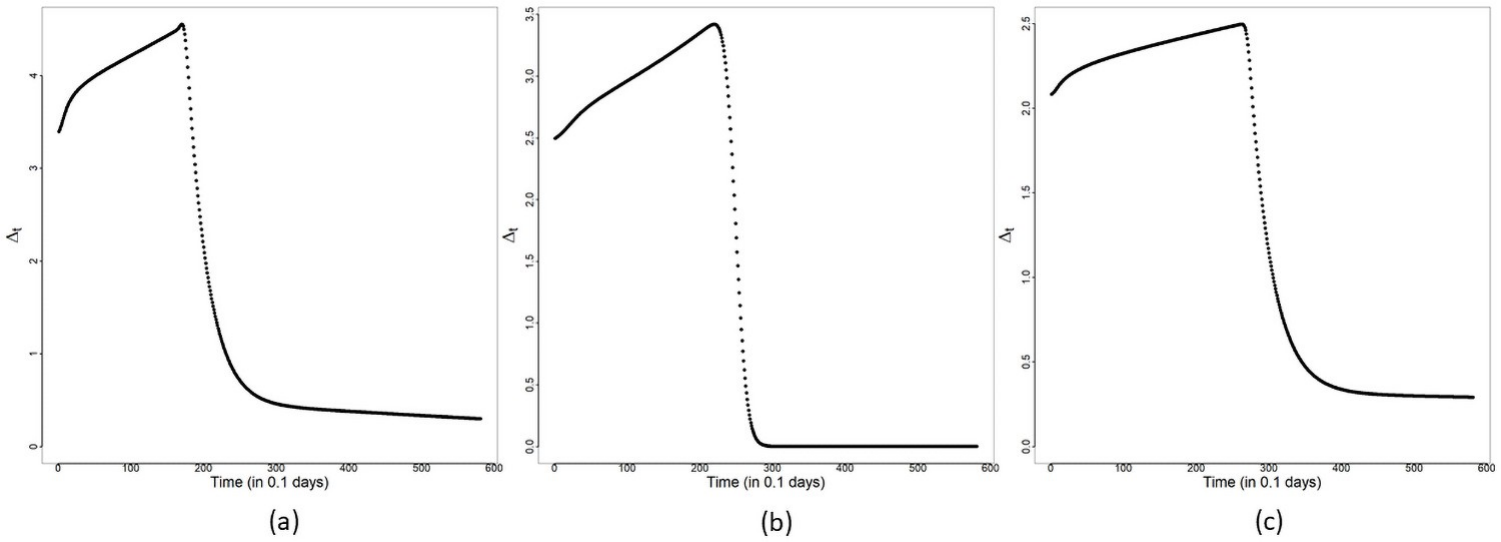
Modified SEIR model: Δ_*t*_. Plots of Δ_*t*_ versus *t*: (a) Malaysia, (b) Selangor and (c) Sarawak.

The plots of Δ_*t*_ versus *t* for Malaysia, Selangor and Sarawak are provided in Fig 9. We note that all three panels in Fig 9 indicate a decay in the transmission rates after T*. The measure Δ_*t*_ is calculated to be approximately 3.39, 2.50 and 2.08 for Malaysia, Selangor and Sarawak, respectively, at the start of $W_{L}$, that is, when *t* = *T*_0_. Starting from *t* = *T*_0_, Δ_*t*_ showed an increase in $W_{L}$, reaching values of4.55, 3.42 and 2.50 at *t* = T*, respectively, for Malaysia, Selangor and Sarawak. Hence, before the MCO was introduced, Selangor achieved a rate of increase higher than that of Sarawak. After T*, Δ_*t*_ for Malaysia, Selangor and Sarawak registered a decay demonstrating the effectiveness of the MCO. Δ_*t*_ declined sharply to a value around0.33, 0.0003 and 0.31, respectively, for Malaysia, Selangor and Sarawak at *t* = T* + 10, and after that, it declined more gradually to its corresponding asymptote. Based on Δ_*t*_, it is seen that the initial transmission rates tend to be higher for areas with a higher population density (comparing Selangor and Sarawak). On the other hand, based on the MAP estimates of *a*_1_ in $W_{R}$ of −1.756 and −0.325 for Selangor and Sarawak, respectively (see Tables 3 and 4), higher population density areas also experience a faster decline in the transmission rates under an effective implementation of the MCO. Although the MAP estimate of *a*_1_ for Malaysia (*a*_1_ = −0.371 from Table 2) is not as negative as it should be, we will show in the next section that a redistribution of cases further improves this estimate of *a*_1_ and brings it closer to that of Selangor.

## Discussion on reporting delays, case redistribution and overdispersed likelihoods

**Fig 10:**
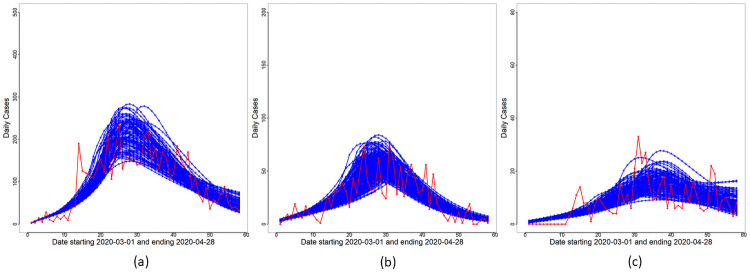
Variation of modelled daily cases. Illustration of the variability of *F*(*t*) from the posterior: Reported cumulative cases (red line), and overlay plots of *F*(*t*) for (a) Malaysia, (b) Selangor and (c) Sarawak.

Fig 10 indicates the presence of outliers that fall outside the limits of variability of the posterior. The most notable outlier is the total number of new cases reported on Day 14 for Malaysia. Generally speaking, such outliers highlight a mismatch between the proposed model and the observed data, and point towards model inadequacy. However, we wish to emphasize that this is not the case here. One key consideration is the effect of delay, that is, whether or not the reported case numbers coincide with the day of testing. It is highly likely that a lag occurred in the reporting of cases since the COVID-19 experience was new to Malaysia. Based on the report [36], it is reasonable to assume that delays in testing and reporting were expected during the initial days of the COVID-19 outbreak in Malaysia. The peak on Day 14 seem to suggest a significant backlog of reporting of cases coupled by the fact that the day was a Monday, the first day of the week.

**Fig 11:**
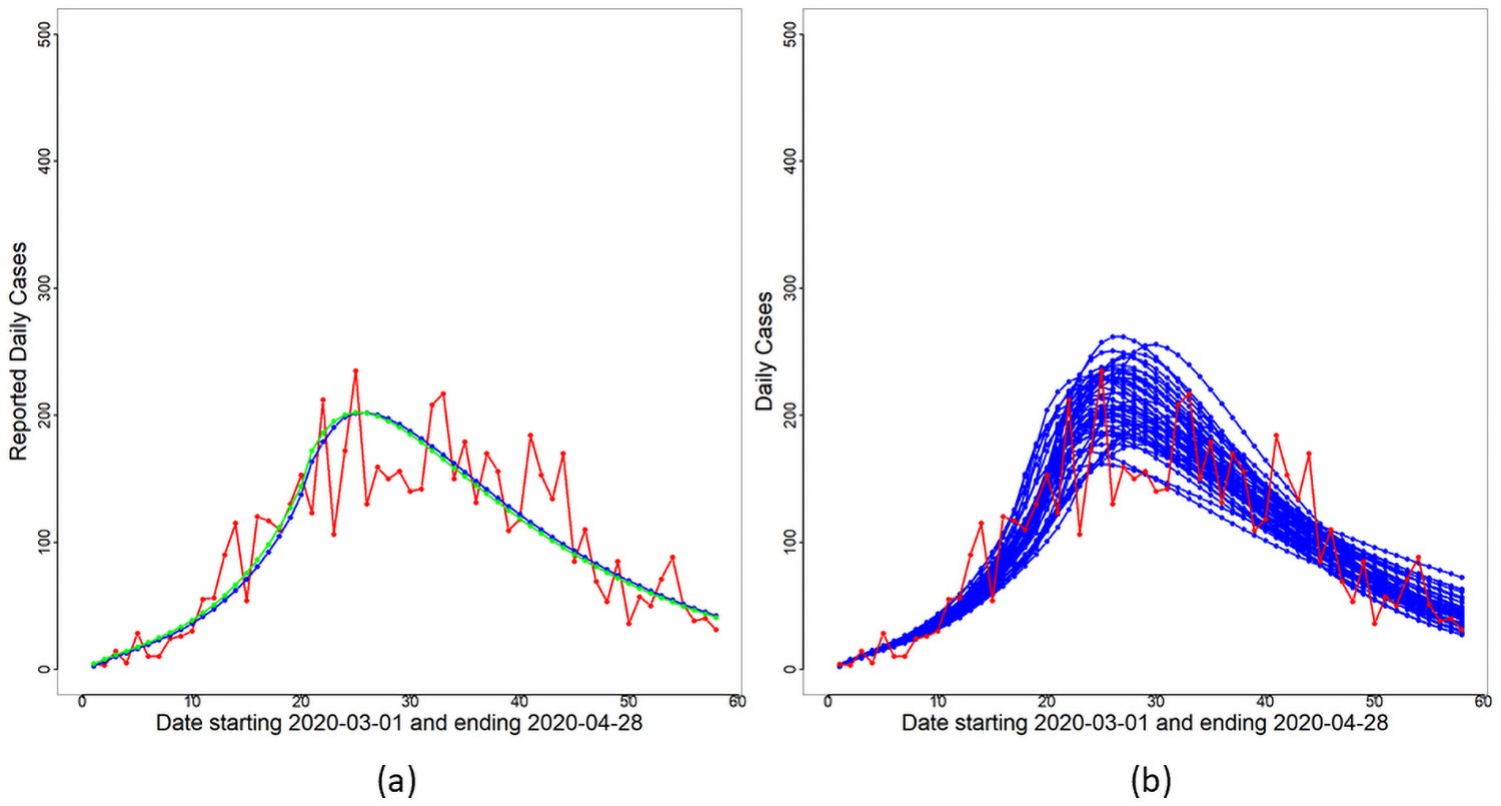
Effect of redistribution. Panel (a) shows the redistributed daily cases and the corresponding best fit curves of *F*(*t*) (blue line) and Δ*R*_*o*_(*t*) (green line) based on ${\hat{\Theta }}_{MAP}$. Panel (b) shows the variability of the fit based on the ensemble set ${\left\{\Theta_{i}^{*}\right\}}_{i=1}^{M}$.

The effects of reporting delays on observed case trajectories and parameter inference are illustrated here based on a simulation study. A delay-in-reporting model based on the multinomial distribution is assumed: Let *X* ∼ *Mult*(*D*_*t*_;*p*_1_, *p*_2_, ⋯, *p*_*K*_), where *X* = (*X*_1_, *X*_2_, ⋯, *X*_*K*_) with *K* = 5 and *X*_*k*_ is the number of cases (out of the total reported cases on day *t*, *D*_*t*_) that is to be redistributed to day *t* − *k* + 1 for *k* = 1, 2, ⋯, *K*. The probabilities *p*_*k*_, *k* = 1, 2, ⋯, *K* are chosen according to a truncated geometric distribution taking values in *k* − 1 for *k* = 1, 2, ⋯, *K* with success probability 0.4. The cases redistribution model is applied to new cases reported from Day 10 until Day 15. The redistributed reported case trajectory, the best fit curves and associated variabilities are shown in Fig 11. Comparing Figs 10(a) and 11(b), one can immediately notice that the reported case numbers in Fig 11(b) are better explained by the variabilities of the underlying model and the negative binomial likelihood. Parameter estimates and credible intervals for the redistributed case numbers are given in Table 5. The new infectious periods are still within the 6–8 day range and are consistent with previously reported literature. The redistribution of case numbers have also reduced the uncertainty around the MAP value of T* = 20: The credible interval for T* in Table 5 is narrower compared to that in Table 2. The MAP estimate of *a*_1_ is now −1.693, which is about halfway between Selangor and Sarawak.

**Table 5:** Summary results for Malaysia (with redistributed cases).

Window	Parameter	MAP	95% Credible Interval
$W_{L}$	*a*_0_	1.787	(1.787, 3.403)
	*w*_*o*_	1.007	(1.007, 1.146)
	*w*_*c*_	1.420	(1.260, 7.011)
	*v*	4.035	(1.170, 4.789)
	*α*	5.95 × 10^−7^	(9.23 × 10^−8^, 9.38 × 10^−7^)
	1/*γ*_*o*_	7.537	(6.707, 8.131)
$W_{R}$	*a*_1_	-1.693	(-2.393, -0.283)
	*a*_2_	13.205	(1.125, 25.933)
	*w*_*o*_	1.319	(1.063, 1.369)
	*w*_*c*_	5.361	(1.660, 6.643)
	*v*	9.550	(1.944, 9.559)
	1/*γ*_*o*_	7.023	(6.224, 7.478)
–	1/*γ*_*c*_	7.615	(7.152, 8.660)
	1/*δ*	7.908	(6.144, 7.996)
	*μ*	0.251	(0.251, 0.974)
	*p*	0.692	(0.529, 0.951)
	*τ*	0.113	(0.103, 0.169)
	*i*_0_	1.899	(0.365, 9.080)
	*e*_0_	270.54	(200.02, 285.09)
	T*	20	(18, 21)

A final point to be discussed is our preference for the negative binomial likelihood compared to the more traditional Poisson likelihood for modelling COVID-19 case numbers. Our initial investigation used the Poisson likelihood for reported case numbers but we found that the underlying model together with the Poisson likelihood was not able to capture inherent variabilities in the observed data. Hence, we opted for the overdispersed negative binomial likelihood which was able to satisfactorily represent the observed data via its overdispersion parameter *τ*. This is evidenced by the variability bands presented in Figs 10 and 11(b) which successfully enclose most of the reported case numbers. This coverage is further improved in Fig 11(b) by a redistribution of delayed cases. We also provide the loglikelihood values corresponding to the Poisson and negative binomial observation models in Table 6 for Malaysia (with original case numbers), Malaysia (with redistributed case numbers), Selangor and Sarawak. Note that the negative binomial loglikelihood values are consistently larger than the Poisson counterparts indicating a better model fit to observed data.

**Table 6:** Loglikelihood values of the NB and poisson likelihoods.

Country/State	Distribution	Log-likelihood values
Malaysia	Negative Binomial	-255.62
	Poisson	-495.45
Malaysia (redistributed)	Negative Binomial	-243.62
	Poisson	-390.01
Selangor	Negative Binomial	-190.98
	Poisson	-276.51
Sarawak	Negative Binomial	-136.30
	Poisson	-160.92

## Conclusion

Quantitative models and assessment of the impacts of the Sri Petaling gathering and implementation of MCO on COVID-19 spread in Malaysia are developed in this paper. The MCO implementation is found to be highly effective in containing (an exponential rise of) the COVID-19 outbreak in Malaysia. The analysis here quantitatively demonstrates how quickly transmission rates fall under effective NPI implementation within a short time period. Higher disease transmission is found in Selangor (a state with higher population density) compared to Sarawak. We also found that under MCO, the decline in transmission was faster in Selangor compared to Sarawak. The rise and fall of disease transmission in Selangor mirrored the national level whereas Sarawak showed a more gradual increase and decrease in COVID-19 transmission. The change points were mostly found to be close to the date of MCO implementation (18th March 2020) although Sarawak exhibited a larger uncertainty around that date due to its gradual and slower increasing and decreasing trends of reported case numbers. Our study developed a new model to represent COVID-19 spread in Malaysia that accounts for heterogeneity and asymptomatic transmissions. We found that reported case numbers in Malaysia exhibited large variabilities which can possibly be attributed to a delay in reporting, particularly during the early stages of the pandemic as the experience with handling COVID-19 was new to the country. Nevertheless, the model developed here together with the overdispersed negative binomial likelihood are able to capture salient features of COVID-19 spread in Malaysia and provide reliable quantitative assessments even under the challenges of limited and delayed data.

## Supporting information

S1 Data (XLSX)

## References

[1] Kementerian Kesihatan Malaysia. KPK Press Statement 25 January 2020—Detection of A New Case Infected by The 2019 Novel Coronavirus (2019-nCoV) in Malaysia; 2020. Press Release. Available from: https://kpkesihatan.com/2020/01/25/kenyataan-akhbar-kpk-25-januari-2020-pengesanan-kes-baharu-yang-disahkan-dijangkiti-2019-novel-coronavirus-2019-ncov-di-malaysia/.

[2] Kementerian Kesihatan Malaysia. KPK Press Statement 25 February 2020—The Latest Situation of the Coronavirus Disease 2019 (COVID-19) Infection in Malaysia; 2020. Available from: https://kpkesihatan.com/2020/02/25/kenyataan-akhbar-kpk-25-februari-2020-situasi-terkini-jangkitan-coronavirus-disease-2019-covid-19-di-malaysia/.

[3] Kementerian Kesihatan Malaysia. KPK Press Statement 27 February 2020—The Latest Situation of the Coronavirus Disease 2019 (COVID-19) Infection in Malaysia; 2020. Available from: https://kpkesihatan.com/2020/02/27/kenyataan-akhbar-kpk-27-februari-2020-situasi-terkini-jangkitan-coronavirus-disease-covid-19-di-malaysia/.

[4] Babulal V, Othman NZ. Sri Petaling Tabligh gathering remains Msia’s largest Covid-19 cluster. New Straits Times. 2020. Available from: https://www.nst.com.my/news/nation/2020/04/583127/sri-petaling-tabligh-gathering-remains-msias-largest-covid-19-cluster.

[5] Kementerian Kesihatan Malaysia. Ministry of Health Malaysia Facebook link dated 9 April 2020. Available from: https://m.facebook.com/kementeriankesihatanmalaysia/photos/a.10151657414821237/10156918887836237/?type=3&source=48&__tn__=EH-R.

[6] Novozhilov AS. On the spread of epidemics in a closed heterogeneous population. Math Biosci. 2008;215(2):177–185. 10.1016/j.mbs.2008.07.010 18722386 PMC2580825

[7] Novozhilov AS. Epidemiological Models With Parametric Heterogeneity: Deterministic Theory for Closed Populations. Mathematical Modelling of Natural Phenomena. 2012;7(3):147–167. 10.1051/mmnp/20127310

[8] Banholzera N, van Weenena E, Kratzwalda B, Seeligera A, Tschernuttera D, Bottrighia P, et al. The estimated impact of non-pharmaceutical interventions on documented cases of COVID-19: A cross-country analysis. medRxiv. 2020;.

[9] Cowling BJ, Ali ST, Ng TWY, Tsang TK, Li JCM, Fong MW, et al. Impact assessment of non-pharmaceutical interventions against coronavirus disease 2019 and influenza in Hong Kong: an observational study. Lancet Public Health. 2020;5:279–88. 10.1016/S2468-2667(20)30090-6 PMC7164922 32311320

[10] Ngonghala CN, Iboi E, Eikenberry S, Scotch M, MacIntyre CR, Bonds MH, et al. Mathematical assessment of the impact of non-pharmaceutical interventions on curtailing the 2019 novel Coronavirus. Mathematical Biosciences. 2020;325:1–15. 10.1016/j.mbs.2020.108364 32360770 PMC7252217

[11] Imai N, Gaythorpe KAM, Abbott S, Bhatia S, van Elslandand Kiesha Prem S, Liu Y, et al. Adoption and impact of non-pharmaceutical interventions for COVID-19. Wellcome Open Research. 2020;. 10.12688/wellcomeopenres.15808.1 32529040 PMC7255913

[12] de Figueiredo M, Codina D, Figueiredo MM, M S, León C. Impact of lockdown on COVID-19 incidence and mortality in China: an interrupted time series study. Bull World Health Organ [Submitted]. 2020;.

[13] Thompsona RN, Stockwind JE, van Gaalene RD, Polonskyf JA, Kamvarg ZN, Demarshh PA, et al. Improved inference of time-varying reproduction numbers during infectious disease outbreaks. Epidemics. 2019;29. 10.1016/j.epidem.2019.100356 PMC7105007 31624039

[14] Al Wahaibi A, Al Manji A, Al Maani A, Al Rawahi B, Al Harthy K, Alyaquobi F, et al. COVID-19 epidemic monitoring after non-pharmaceutical interventions: The use of time-varying reproduction number in a country with a large migrant population. International Journal of Infectious Diseases. 2020;99:466–472. 10.1016/j.ijid.2020.08.039 32829052 PMC7439014

[15] Giordano G, Blanchini F, Bruno R, Colaneri P, Di Filippo A, Di Matteo A, et al. Modelling the COVID-19 epidemic and implementation of population-wide interventions in Italy. Nature Medicine. 2020; p. 1–6. 10.1038/s41591-020-0883-7 32322102 PMC7175834

[16] Hu FC. The Estimated Time-Varying Reproduction Numbers during the Ongoing Pandemic of the Coronavirus Disease 2019 (COVID-19) in 12 Selected Countries outside China. medRxiv. 2020;.

[17] Kementerian Kesihatan Malaysia. Annexure 2: Management of Suspected, Probable and Conformed COVID-19 Cases; 2020. Available from: http://covid-19.moh.gov.my/garis-panduan/garis-panduan-kkm/Annex_2_Management_of_Suspected,_Probable_and_Confirmed_COVID_07102020.pdf.

[18] Yu P, Zhu J, Zhang Z, Han Y. A Familial Cluster of Infection Associated With the 2019 Novel Coronavirus Indicating Possible Person-to-Person Transmission During the Incubation Period. The Journal of Infectious Diseases. 2020;221(11):1757–1761. 10.1093/infdis/jiaa077 32067043 PMC7107453

[19] Wei WE, Li Z, Chiew CJ, Yong SE, Toh MP, Lee VJ. Presymptomatic Transmission of SARS-CoV-2—Singapore, January 23—March 16, 2020. MMWR Morb Mortal Wkly Rep 2020. 2020;69:411–415. 10.15585/mmwr.mm6914e1 32271722 PMC7147908

[20] Kimball A, Hatfield K, Arons M, James A, Taylor J, Spicer K, et al. Asymptomatic and Presymptomatic SARS-CoV-2 Infections in Residents of a Long-Term Care Skilled Nursing Facility—King County, Washington, March 2020. MMWR Morb Mortal Wkly Rep 2020. 2020;69:377–381. 10.15585/mmwr.mm6913e1 PMC7119514 32240128

[21] Blackwood JC, Childs LM. An introduction to compartmental modeling for the budding infectious disease modeler. Letters in Biomathematics. 2018;5(1):195–221. 10.30707/LiB5.1Blackwood

[22] Kermack WO, McKendrick AG. A contribution to the mathematical theory of epidemics. Proceedings of the royal society of london Series A, Containing papers of a mathematical and physical character. 1927;115(772):700–721.

[23] Anguloa MT, Velasco-Hernandez JX. Robust qualitative estimation of time-varying contact rates in uncertain epidemics. Epidemics. 2018;24:98–104. 10.1016/j.epidem.2018.03.001 29567063

[24] Dehning J, Zierenberg J, Spitzner P, Wibral M, Neto JP, Wilczek M, et al. Inferring change points in the spread of COVID-19 reveals the effectiveness of interventions. Science 10 7 2020; 369 (6500), eabb9789. 10.1126/science.abb9789 32414780 PMC7239331

[25] Romero-Severson EO, Hengartner N, Meadors G, and Ke R. Change in global transmission rates of COVID-19 through May 6 2020. PLOS One. 2020. 10.1371/journal.pone.0236776 32760158 PMC7410207

[26] Altahir AA, Mathur N, Thiruchelvam L, Abro GEM, Radzi SSM, Dass SC, et al. Modeling the Impact of Lock-down on COVID-19 Spread in Malaysia. bioRxiv. 2020. 10.1101/2020.07.17.208371

[27] Becker NG, Glass K, Barnes B, Caley P, Philp D, McCaw J, et al. Using Mathematical Models to Assess Responses to an Outbreak of an Emerged Viral Respiratory Disease. Final Report to the Australian Government Department of Health and Ageing National Centre for Epidemiology and Population Health, Australian National University. 2006;.

[28] Hwang J, Park H, Jung J, Kim SH, Kim N. Basic and effective reproduction numbers of COVID-19 cases in South Korea excluding Sincheonji cases. medRxiv. 2020;.

[29] Organization WH, et al. Report of the WHO–China Joint Mission on Coronavirus Disease 2019 (COVID-19), 16–24 February 2020; 2020. Available from: https://www.who.int/docs/default-source/coronaviruse/who-china-joint-mission-on-covid-19-final-report.pdf.

[30] Frasso G, Lambert P. Bayesian inference in an extended SEIR model with nonparametric disease transmission rate: an application to the Ebola epidemic in Sierra Leone. Biostatistics. 2019;17(4):779–792. 10.1093/biostatistics/kxw027 27324411

[31] Xu X, Kypraios T, O‘Neill P. Bayesian non-parametric inference for stochastic epidemic models using Gaussian Processes. Biostatistics. 2016;17(4):619–633. 10.1093/biostatistics/kxw011 26993062 PMC5031942

[32] World Health Organization. Coronavirus disease 2019 (COVID-19) Situation Report 73; 2020. Available from: https://www.who.int/docs/default-source/coronaviruse/situation-reports/20200402-sitrep-73-covid-19.pdf.

[33] Backer JA, Klinkenberg D, Wallinga J. Incubation period of 2019 novel coronavirus (2019-nCoV) infections among travellers from Wuhan, China, 20–28 January 2020. Eurosurveillance. 2020;25(5):2000062. 10.2807/1560-7917.ES.2020.25.5.2000062 32046819 PMC7014672

[34] Petersen E, Koopmans M, Go U, Hamer DH, Petrosillo N, Castelli F, et al. Comparing SARS-CoV-2 with SARS-CoV and influenza pandemics. The Lancet infectious diseases. 2020;. 10.1016/S1473-3099(20)30484-9 32628905 PMC7333991

[35] Efron B, Two Modeling Strategies for Empirical Bayes Estimation. Statistical Science, 2014; 29 (2). 10.1214/13-STS455 25324592 PMC4196219

[36] Emil Zainul. Malaysia to boost virus testing with S Korean test kits; 2020. Available from: https://www.theedgemarkets.com/article/malaysia-boost-virus-testing-s-korean-test-kits.

